# Genetic Structure Analysis of 155 Transboundary and Local Populations of Cattle (*Bos taurus*, *Bos indicus* and *Bos grunniens*) Based on STR Markers

**DOI:** 10.3390/ijms24055061

**Published:** 2023-03-06

**Authors:** Evgenia Solodneva, Gulnara Svishcheva, Rodion Smolnikov, Sergey Bazhenov, Evgenii Konorov, Vera Mukhina, Yurii Stolpovsky

**Affiliations:** 1 Vavilov Institute of General Genetics, Russian Academy of Sciences, 119333 Moscow, Russia; 2 Institute of Cytology and Genetics, Siberian Branch of the Russian Academy of Sciences, 630090 Novosibirsk, Russia; 3 Gorbatov Federal Research Center for Food Systems, Russian Academy of Sciences, 109316 Moscow, Russia

**Keywords:** STR analysis, cattle, local breeds, *Bos taurus*, *Bos grunniens*, *Bos indicus*, phylogenetic relationship

## Abstract

Every week, 1–2 breeds of farm animals, including local cattle, disappear in the world. As the keepers of rare allelic variants, native breeds potentially expand the range of genetic solutions to possible problems of the future, which means that the study of the genetic structure of these breeds is an urgent task. Providing nomadic herders with valuable resources necessary for life, domestic yaks have also become an important object of study. In order to determine the population genetic characteristics, and clarify the phylogenetic relationships of modern representatives of 155 cattle populations from different regions of the world, we collected a large set of STR data (10,250 individuals), including unique native cattle, 12 yak populations from Russia, Mongolia and Kyrgyzstan, as well as zebu breeds. Estimation of main population genetic parameters, phylogenetic analysis, principal component analysis and Bayesian cluster analysis allowed us to refine genetic structure and provided insights in relationships of native populations, transboundary breeds and populations of domestic yak. Our results can find practical application in conservation programs of endangered breeds, as well as become the basis for future fundamental research.

## 1. Introduction

The erosion and extinction of the genetic resources of domesticated animal species is a global problem (FAO 2021) [1]. According to Simianer [2], 1–2 breeds of farm animals including aboriginal cattle disappear weekly. Such cattle are the custodians of rare allelic variants of genes responsible for adaptation to extreme environmental conditions, resistance to diseases and for the ability to obtain maximum energy from a meager diet. Alleles that are neutral for habitual environmental conditions, accumulated in the genomes of local cattle, can become useful in case of a rapid change in environmental conditions. Thus, biodiversity conservation expands the range of genetic solutions to potential problems of the future. Population genetic studies, revealing the structure of aboriginal breed populations, demonstrate their uniqueness and promote other researchers to look for causal variants of adaptation and health genes. In addition, the assessment of inbreeding level, expected and observed heterozygosity, deviation from the Hardy–Weinberg equilibrium and other characteristics help to develop right conservation strategy for breeds that are on the verge of extinction. The availability of genotyping methods has contributed to an increase in the number of studies in the field of assessing the genetic diversity of cattle [3,4,5,6]. The genetic individuality of some cattle breeds, in particular from Russia, has been demonstrated [7,8,9]. Rare allelic variants of genes responsible for adaptation, disease resistance and productivity have been found in the genome of the Yakut, Kholmogory and Yaroslavl breeds [10,11,12]. The origin of Russian cattle breeds has been discussed in a number of major studies [3,13,14,15,16], but remains controversial for some breeds. The relevance of conducting additional research on the Russian local cattle breeds of Asian origin in the context of a wider range of *Bos indicus* breeds has been discussed, for example, in the work of Yurchenko et al. [3]. The authors of another large study [13] have indicated the need to continue the study of the Kholmogory and Kalmyk breeds.

The domestic yak (*Bos grunniens*) is a valuable resource for nomadic herders and an important object to study. Recent studies of *Bos grunniens* diversity demonstrated that STR (short tandem repeat) markers used to explore the genetic diversity of cattle [17,18] are well-suited for population analysis of domestic yaks. So far, STR analysis was performed for yak populations from Mongolia [19], China [20], Bhutan [21], India [22], Switzerland [23] and Russia [17]. Most population studies related to yaks living in Russia focus on their hybrids [24,25,26] whereas the rest of the studies is usually limited to a several dozen yak individuals. This limitations do not allow us to draw a conclusion about the true genetic diversity of yaks from Russia [19,27]. Structural analysis of domestic yak populations against the background of a large sample of transboundary and local breeds allowed us to reveal the mutual introgression of genomes.

To study the parameters of genetic diversity and hybridization, as well as evolutionary processes, most studies use SNP (single nucleotide polymorphism) and STR markers; mitochondrial DNA and the Y-chromosome variation. Microsatellites have long established themselves as reliable markers. They are evenly distributed throughout the eukaryotic genome [28] and are highly polymorphic due to the variations in the number of repeating units [29]. Li et al. reported that within-breed variability results (89%) based on SNP markers were consistent with those obtained using STR markers [30].

In order to determine the population genetic characteristics, and clarify the phylogenetic relationships for 155 cattle populations from different regions of the world, we analysed genetic variability of 12 highly variable microsatellite loci included in the panel recommended by ISAG-FAO [31]. The studied populations include unique native cattle from different regions of the world, 12 yak populations from Russia, Mongolia and Kyrgyzstan, as well as zebu breeds. During the study, the main genetic characteristics of the studied populations were calculated: mean number of alleles (Na), effective number of alleles (Ne), allelic frequencies (AF), allelic diversity parameters, observed (Ho) and expected (He) heterozygosity, inbreeding coefficient (Fis), Hardy–Weinberg equilibrium (HWE) and polymorphism information content (PIC). Using the neighbor-joining method, a phylogenetic tree was constructed based on the genetic distance matrix Da. Moreover, principal component analysis was used to analyse phylogenetic relationships. Using Bayesian clustering analysis we analysed the genetic structure of the studied animal samples. Taken together, these results lead to several important clarifications regarding the genetic structure of the populations of native cattle and domestic yak.

## 2. Results

Subchapters in Results and Discussion are subdivided into breed groups for convenience of narration and perception of the text.

### 2.1. Genetic Variability

For the 12 microsatellites analysed, 215 alleles were detected, with 12 (Bm1824) to 26 (Tgla122) per locus. The mean number of alleles per locus across all samples was 17.9. The average percentage of the total number of alleles observed in the locus varied from 19.55% (Altai-Rus) to 63.76% (Baladi) (Table A1). We calculated allele frequencies (AF) and the PIC values (Table S1) as a measure of the amount of information that can be recovered from a genetic marker.

High AF values (AF > 0.8) were obtained for five loci: locus Eth10 with alleles 217 (Charolais and Romagnola), 219 (Guabalá and Brava de Lide) and 221 (Vaca Palmera); locus Bm1824 with alleles 180 (Scottish Highlander and Muturu) and 182 (Heck Cattle); locus Csrm60 with allele 102 (Scottish Highlander); locus Eth225 with allele 140 (Guabalá); locus Sps115 with alleles 248 (Belgian Blue, Groningen Whiteheaded, Heck Cattle, Dutch Belted, Crioulo Lageano, Lidia, Marismeña, Mallorquina, Barrosã, Brava de Lide, Cachena, Garvonesa, Preta, Baladi, Menoufis, Bafatá, Gabú and Ankole-Watusi) and 256 (Yakut); and locus Eth3 with allele 117 (Romagnola, Jersey).

For each locus and each sample, the PIC values were estimated on the basis of the number and frequency of alleles at the locus. The mean PIC value appeared to be 0.795. The highest polymorphism levels were obtained for Tgla122 (PIC = 0.897), Csrm60 (PIC = 0.888), Bm1824 (PIC = 0.856) and ilsts006 (PIC = 0.842).

The genetic individuality of a breed may be defined by private (potentially breed-specific) alleles. Out of 215 alleles in 10,250 animals genotyped, 23 alleles were private. All the described private alleles were discovered in the genomes of local cattle breeds (from Cuba, Kyrgyzstan, Brazil, Angola, Italy, India, Russia, Egypt, Spain, Nigeria, Zambia and Colombia) and various populations of domestic yak. The remaining private alleles were revealed in the genomes of various populations of domestic yak. The highest numbers of private alleles equal to 3 were found in representatives of the Gyr breed from India, Siboney breed from Cuba, and in the domestic yak population. Within these particular breeds the largest number of private alleles per locus equal to 4 was detected for Tgla227 (Table 1). The largest numbers of private alleles adjusted for sample size were in the Gyr, Red Bororo and Siboney native cattle (25%, 7.7% and 6%, respectively, of their total number). The presence of private microsatellite alleles with frequencies above 0.01 in the native cattle breeds suggests that each of these breeds most likely has a unique gene pool.

Genetic variability in each animal sample was studied in terms of the number of alleles (A), allelic richness (Ar), observed (Ho) and expected (He) heterozygosity, inbreeding coefficient (Fis) and *p*-value for the chi-square test of the Hardy–Weinberg equilibrium (Table A1). Ar varied from 3.22 (Altai-Rus) to 6.57 (Baladi), with a mean of 5.1. Allelic richness is more useful in identifying genetic bottlenecks comparing to expected heterozygosity because of its sensitivity to the loss of rare alleles and differences in sample size. We explored the relationship between Ar and He and revealed a significant correlation (0.93) with the determination coefficient R2 = 0.88 (*p*-value = 5 × 10^−73^). The observed and expected heterozygosity values were compared using the Bartlett test. The results obtained showed that there was a difference between the mean Ho and He values, and the pooled sample deviates from the Hardy–Weinberg equilibrium (*p*-value = 0.0015). High Ho values (0.81) were found for Criollo Baja California and Criollo Pilcomayo and the lowest (0.57) for Aryg-Khem-Rus.

We calculated the inbreeding coefficient (Fis) for each sample as Fis = (He − Ho)/He. High Fis values point to a decreased heterozygosity of the microsatellites due to inbreeding. A very severe deficiency of heterozygotes was observed in two samples: Pineywoods (Fis = 0.166) and Criollo Yacumeño (Fis = 0.162). The Fis values were below zero, and the prevalence of heterozygotes were found in 60 breeds; the lowest ones are Sindi, Altai-Rus and Blanco Orejinegro. This implies that mating within the breeding farms is random and nonassortative, and no inbreeding occurs. For the other breeds, we observed neither strong excess nor strong deficiency of heterozygotes. The mean Fis value in the pooled sample is 0.003.

### 2.2. Phylogenetic Analysis and Principal Component Analysis

#### 2.2.1. Domestic Yak (*Bos grunniens*)

**Figure 1:**
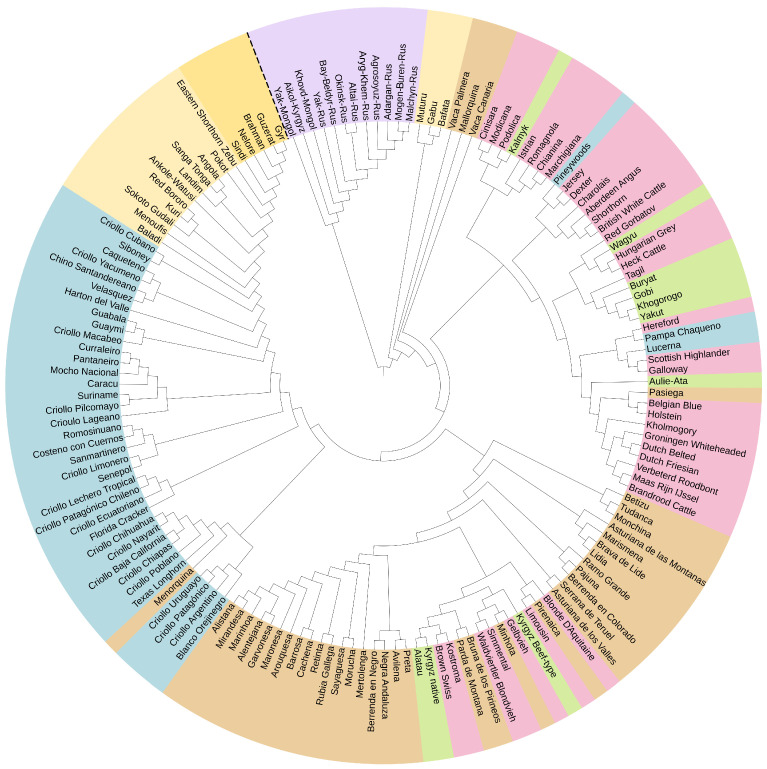
Phylogenetic tree constructed by the neighbor-joining algorithm using Da genetic distances. The circular range is represented by 7 main clusters (breed group): purple (Domestic yak), yellow (Zebu), light yellow (African), beige (Iberian), pink (European), light green (Asian) and blue (Creole). The root of the tree was selected automatically for better visualisation and represents the basic division between modern yaks and the rest.

**Figure 2:**
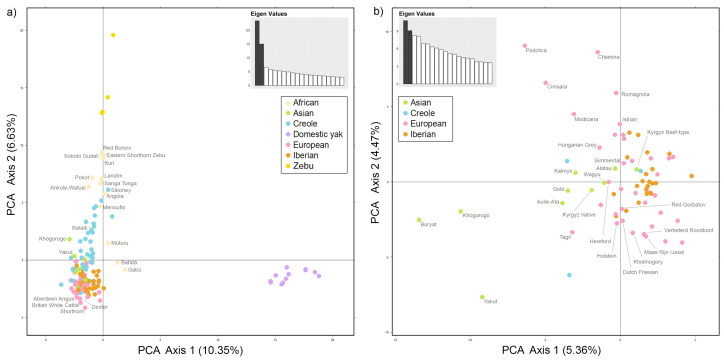
Principal component analysis. Spatial representation of the genetic distances between the analysed breeds along the first two axes obtained by factor matching analysis based on microsatellite data. The values in parentheses on both axes represent the percentage contribution of each axis to the total inertia. The colours represent belonging to a breed group, as shown in the figure. The names of some breeds are given. (**a**) PCA results for 155 populations. (**b**) PCA results for a sample of predominantly European and Asian breeds from Cinisara to Alatau (according to the dendrogram).

Both on the phylogenetic tree (Figure 1) and on the PCA plot (Figure 2a), the population of yaks (*Bos grunniens*) separated away from the rest of the breeds, which is consistent with the modern idea of the cattle phylogeny.

#### 2.2.2. European Breed Group (*Bos taurus*)

Most European breeds are grouped together in a distinct cluster on a phylogenetic tree, with only a few breeds from other breed groups included. Among them, there was also a Kalmyk breed from the Asian breed group, united with Podolica. PCA (Figure 2b) showed that the Kalmyk breed is located between the European and Asian breeds, which may indicate the presence of common ancestors, with representatives both from the Podolian breeds and breeds of the Turano-Mongolian root. To estimate the level of genetic differentiation between the populations of the Asian breed group, which includes Kalmyk cattle and Grey cattle breeds of the European breed group, pairwise Fst values were calculated (Figure S1 and Table S2). Several levels of significance (*p*-values = 0.05; 0.01 and 0.005) were examined for genetic differentiation. Fst analysis demonstrates that studied samples are significantly distanced from each other. The greatest divergence was noted between the Japanese Wagyu and the Italian Marchigiana (Fst = 0.1386, *p*-value = 0.001). Representatives of the Kyrgyz native and the Alatau breeds were most closely related to each other (Fst = 0.0050, *p*-value = 0.4750), which is consistent with the results of the previous study [8], as well as the origin history of the Alatau breed [32]. Analysing the relationship of Kalmyk cattle with the Asian and the Grey cattle breeds, we found that the least genetic differentiation is observed with the Buryat (Fst = 0.0077, *p*-value = 0.1239), the Podolica (Fst = 0.0128, *p*-value = 0.0370) and the Kyrgyz native breeds (Fst = 0.0137, *p*-value = 0.0060). At the same time, nonsignificant or weak genetic differentiation of Buryat cattle with most of the studied breeds should be noted. The greatest differentiation of Kalmyk cattle was observed with the Yakut (Fst = 0.0730, *p*-value = 0.001), Chianina (Fst = 0.0576, *p*-value = 0.001) and Wagyu (Fst = 0.0526, *p*-value = 0.001) breeds. Thus, the results of the Fst analysis testify in favor of the presence of common ancestors of Kalmyk cattle, both with the representatives of the Grey root and with the representatives of the Turano-Mongolian root. The Istrian breed from the Istrian peninsula also entered the cluster of Italian breeds, which is probably due to the territorial proximity and possible hybridization.

Next to Italian breeds there is a cluster of the United Kingdom cattle: Dexter, Jersey, Aberdeen Angus, Shorthorn and British White Cattle. The French meat breed Charolais joined this group. Its clustering near the Aberdeen Angus breed can be explained by known cases of crossing representatives of these two breeds. Another large cluster is formed by Dutch breeds, including Brandrood Cattle, Maas Rijn IJssel, Verbeterd Roodbont, Dutch Friesian, Dutch Belted and Groningen Whiteheaded. It is noteworthy that this group is joined by a breed from Russia—Kholmogory. The history of its breeding dates back to the 17th century and is noted for crossing with “Dutch cattle” [33]. The Red Gorbatov and Tagil breeds fell into a cluster of predominantly Asian breeds, within the European cluster. Such clustering of the Red Gorbatov breed is probably due to the absence in the studied sample of breeds that participated in the creation of the Red Gorbatov cattle. A small cluster of European and Asian breeds turned out to be inside the main cluster of the Iberian breed group. In particular, the Kostroma breed got here, which, as expected, united with Brown Swiss, the one of its ancestral forms.

#### 2.2.3. Asian Breed Group (*Bos taurus*)

The majority of Asian breeds were included in the European breeds sector. Breeds from the Turano-Mongolian root Buryat, Gobi, Khogorogo and Yakut united in one cluster, and Wagyu proved to be closer to European breeds. It is known that Wagyu is experiencing European influence, including a British one [34]. Aulie-Ata is adjacent to the Dutch breeds cluster. It is known that Aulie-Ata was bred by crossing local cattle with Dutch [8].

Three more breeds of the Turano-Mongolian root (Alatau, Kyrgyz Beef-type and Kyrgyz native), together with some European breeds, were localized in the Iberian cluster. In close proximity to Alatau were Kyrgyz native, Kostroma and Brown Swiss. All three breeds were used in the breeding of the Alatau breed [32].

#### 2.2.4. Iberian and Creole Breed Group (*Bos taurus*)

Iberian breeds form separate cluster near the European breed group. The territory of the Iberian Peninsula could act as a contact zone between the African and European continents through the Strait of Gibraltar [35]. The Vaca Canaria and Vaca Palmera breeds from the Canary Islands and the Mallorquina from the Balearic Islands split off from the general Iberian cluster and can be found alongside the African breeds Gabú, Bafatá and Muturu. Such clustering may indicate a significant influence of African cattle on the listed breeds. Two other Iberian breeds were also outside the Iberian breed group. One of them, Pasiega, was in the same cluster as the Holstein breed. Similar clustering was also observed in the work of Mastrangelo et al. [14]. Bruna de los Pirineos and Parda de Montaña were in the same cluster as Brown Swiss and Pirenaica, which is consistent with the history of their origin from the latter two breeds [36,37,38]. The Creole breeds together with African cattle and Zebu formed a sister clade to the Iberian breed group, which is explained by the history of the emergence of Creole cattle, described in detail in a number of works [39,40,41,42,43]. Three Creole breeds are among the European breed group: Pampa Chaqueno, Lucerna and Pineywoods. The authors of the study of Y-haplotypes of cattle [44] confirm crossing with British cattle, namely Pampa Chaqueno with Hereford, whose joint clustering was also noted in our study. The relationship between European and Creole breeds is also described in Decker et al. [45].

#### 2.2.5. Zebu Breed Group (*Bos indicus*)

All five breeds of this group formed a common cluster on the phylogenetic tree near the African breed group, which is explained by the high proportion of *Bos indicus* in the genomes of African breeds. On the PCA plot, zebu breeds also formed a separate group. The farthest of all was the breed of Indian zebu Gyr.

#### 2.2.6. African Breed Group (African Humpless *Bos taurus*, Humped *Bos indicus*, A.h. *Bos taurus* × Humped *Bos indicus*)

According to the classifications available in the literature [46,47,48], the presented breeds of indigenous African cattle can be divided into several groups: humpless *Bos taurus*, humped *Bos indicus*, sanga (African humpless *Bos taurus* × humped *Bos indicus* hybrid) and zenga (sanga×zebu hybrid). The Muturu, Gabu and Bafata breeds living on the west coast of Africa belong to the African phylum *Bos taurus* [49]. On the phylogenetic tree, the listed breeds form a separate cluster located next to the Iberian insular breeds. On the PCA plot, Muturu, Gabu and Bafata are also distant from the African breed group and are located next to the Creole and Iberian breeds. There is also a large cluster of predominantly European breeds nearby. Some works have shown a common origin of Muturu and South European taurine, which may partially explain this co-localization on the phylogenetic tree [50]. Baladi and Menoufis are adjacent to the Creole breed group cluster, which confirms the previously described genetic link [51]. On the dendrogram, the Eastern Shorthorn Zebu and Pokot are closest to the zebu cluster. Both breeds are bred in Kenya. The territorial proximity and colocation on the phylogenetic tree may indicate the possible interbreeding of representatives of the described breeds. Eastern Shorthorn Zebu is a hybrid of Asian zebu and African taurine cattle according to microsatellite analysis [52,53]. A significant part of the Eastern Shorthorn Zebu genome remains common with the Nelore zebu breed [54]. The Kuri breed belongs to the humpless cattle of West Africa [46]. At the same time, on the phylogenetic tree, Kuri clustered with two breeds of the African zebu. It is known from the literature that interbreeding with zebu is currently taking place, in particular with the M’Bororo breed [46].

### 2.3. Bayesian Cluster Analysis

**Figure 3:**
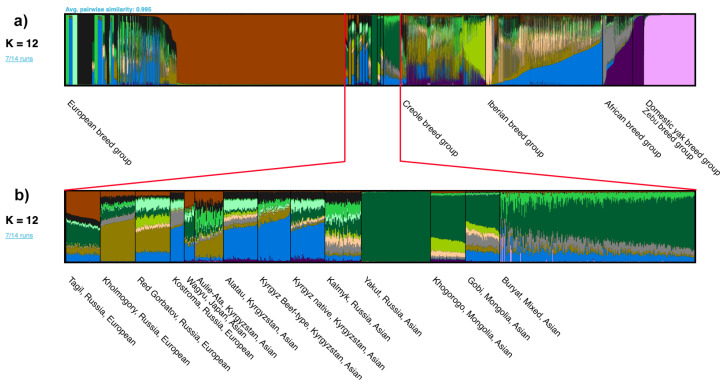
The evaluation of the structure of each individual from the studied populations for K = 12. Each individual is represented by a separate column. Colour identification reflects the proportion of 12 estimated ancestral populations in the genome of a particular individual. (**a**) Structure of 10,250 individuals belonging to seven breed groups (European, Asian, Creole, Iberian, African, Zebu and Domestic yak). (**b**) Structure of individuals belonging to populations of the Asian breed group, indicating the country and 4 Russian breeds from the European breed group.

**Figure 4:**
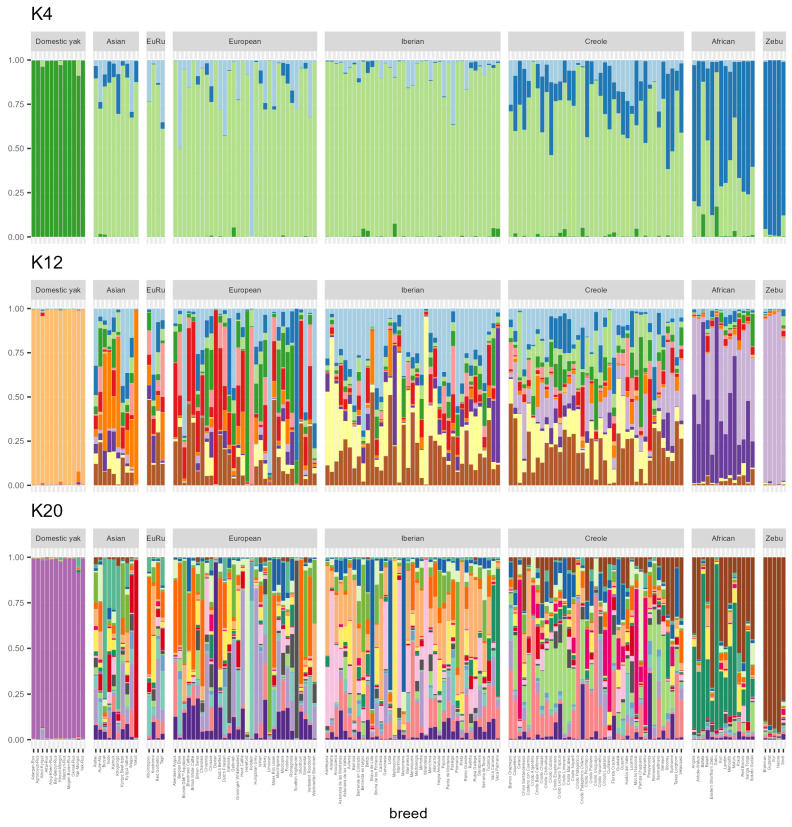
Cluster analysis of 155 cattle populations calculated from microsatellite data in the STRUCTURE program. Each breed is represented by a vertical column divided into K colours, according to the number of estimated ancestral populations. Size of the colour segment is proportional to the contribution of a particular ancestral population to the genome of the breed in question. The results for K = 4, 12 and 20 are presented. The values of K from 2 to 20 are shown in the additional Figure S2.

Figure 3 presents the results for each individual, and Figure 4 contains the results of the cluster analysis for each breed. Generally these results correlate well with PCA and phylogenetic analysis. As expected, domestic yaks are first to form a separate group, at K = 2. Next, at K = 3, the Holstein breed was isolated into a separate group. Among the Russian breeds, at this stage, the Yakut, Kalmyk and Kostroma breeds showed the least influence of the Holstein breed. At K = 4 (Figure 4), a zebu cluster was manifested. At this stag its influence was more widespread on African cattle breeds, Creole cattle and Asian breeds. In addition, the introgression of *Bos taurus* genes into the genomes of domestic yaks and zebu was recorded. The subsequent differentiation of breeds (K = 6) led to the isolation of the Yakut breed. A similar divergence of Yakut cattle is noted in these works: Li et al. [30], Iso-Touru et al. [11], Yurchenko et al. [3] and Li and Kantanen [55]. The Dexter breed is allocated in an independent column at K = 7. At K = 9, the Hereford breed is grouped separately, as well as the Iberian Mirandesa breed; the genomic component of African groups (Figure S2) begins to manifest itself. The optimal number of clusters according to the Evanno method is 3 (Figure S3).

#### 2.3.1. Domestic Yak (*Bos grunniens*)

According to the results of cluster analysis at K = 12, the domestic yak populations from Mongolia contain a Turano-Mongolian component. At the same time, we noted the influence of European breeds in the Aikol yak breed from Kyrgyzstan. Earlier, it was found that 1.3% of the genes of Mongolian yaks were inherited from bovine ancestors [56]. The identified genes are involved in the development and functioning of the nervous system. We probably observe this introgression in our study. We also noticed the presence of the genomic component of yaks in some cattle breeds from different breed groups at K = 4 (Figure 4), which is especially large in two breeds from Africa, Gabú and Bafatá. The same breeds are less affected by *Bos indicus*. Among the animals of the Asian breed group we revealed another trace of probable introgression from the yak, in particular in the Buryat and Aulie-Ata breeds. Referring to Figure 3, which presents the results of cluster analysis for individuals, seven individuals of the Buryat breed and one individual of the Aulie-Ata have significant contribution of the yak genomic component, which indicates recent hybridization events.

#### 2.3.2. European Breed Group (*Bos taurus*)

As K values increased, European breeds were separated into their own clusters. In particular, at K = 12, the following breeds formed their own clusters: Holstein, Dexter, Hereford, Limousin and Scottish Highlander. At K = 10 (Figure S2), all studied Mediterranean breeds are influenced to varying degrees by the African group, which at K = 20 (Figure 4) remains well-distinguishable in the Italian breeds Romagnola, Modicana and Cinisara. It is noted in the literature that cattle of the Middle East and Africa could leave their mark in the genomes of Mediterranean breeds [42].

#### 2.3.3. Asian Breed Group (*Bos taurus*)

At K = 12, the predominance of bright orange colour in the cluster of Asian breeds is clearly visible. We assume that this colour indicates the Asian, probably Turano-Mongolian, origin (Figure 4). The largest contribution of this component can be traced in the Yakut breed from Russia (about 97%), which is consistent with the results of previous studies [3,11,30]. Next in percentage are the Khogorogo from Mongolia and the Buryat from Russia. The smallest contribution of the Turano-Mongolian component among the breeds of the Asian cluster was noted in the Kyrgyz beef-type and Alatau breeds from Kyrgyzstan. Increasing the K values to 20 (Figure 4) led to the division of the supposed Turano-Mongolian component into two clusters. The first cluster united the Buryat and Khogorogo breeds, and the second one was represented by the Yakut breed. A similar differentiation was also demonstrated by the principal component analysis (PCA) results shown in Figure 2b. The genetic affinity of the Buryat breed from Russia and Khogorogo from Mongolia reflects a closely interwoven history of the Buryat and Mongolian people. Other breeds included in the Asian breed group showed involvement in one of the presented groups or included both components. The hypothesis of the origin of East Asian cattle from two different ancestors of *Bos taurus* was previously described by Chen et al. [57].

The results of our structural analysis also indicate the presence of an Asian component in the genomes of some Italian breeds. In particular, at K = 12, among the Italian breeds, the Podolian Podolica, the Sicilian Modicana and Cinisara demonstrated the greatest contribution of the Asian ancestry. At K = 20, the putative Asian component in Podolica showed commonality with the ancestors of Buryat (Russia) and Khogorogo (Mongolia). At the same time, on the PCA plot, the Podolica grouped with other Italian breeds, away from the Asian breed group. Structural analysis of the Asian breed group at K = 20 shows that most of the populations have a clearly visible genetic component of European breeds.

#### 2.3.4. Iberian and Creole Breed Group (*Bos taurus*)

Structural analysis demonstrated that at K = 20, the breed Marismeña (Figure 4) joined the Brava de Lide and Lidia group in alignment with our phylogenetic tree (Figure 1). Until recently, the origin of Marismeña was in doubt, and several hypotheses have been put forward, including the origin from the crossing of local cattle with Lidia bulls.

#### 2.3.5. Zebu Breed Group (*Bos indicus*)

Zebu breeds formed a separate cluster at K = 4 (Figure 4). The influence of *Bos indicus* is more widespread on the African and Creole breed group, which is consistent with the previously described history of their origin. The exception was made by several Creole breeds, where zebu influence was barely perceptible. Such breeds include Criollo Argentina (Argentina), Criollo Patagónico (Argentina), Criollo Patagónico Chileno (Chile) and Romosinuano (Colombia). A previous study showed the absence of male-mediated introgression of *Bos indicus* into the genomes of Argentine breeds [44]. The West African breeds Gabú, Bafatá, and Muturu experience the least impact of *Bos indicus* in the African breed cluster. They were previously separated from the African breed group and formed a separate cluster on both the phylogenetic tree and the PCA plot. These breeds belong to the African type *Bos taurus*. Ankole-Watusi and Eastern Shorthorn Zebu showed the highest percentage of indicine ancestry. At K = 4, all representatives of the Asian breed group demonstrate in their structure a trace of *Bos indicus*, best discernible in the Khogorogo breed from Mongolia. Earlier, the contribution of indicine ancestors to the genome of the Turano-Mongolian breeds [3] was described in the literature. Animals from European and Iberian breed group, on the contrary, mostly showed no or very little zebu ancestry, which is consistent with previous studies [34,58]. At K = 4 (Figure 4) impact *Bos indicus* genome is most prominent in the following European breeds: Italian Cinisara, Modicana, Chianina, Romagnola and Podolica, Croatian Istrian, Swiss Simmental and Brown Swiss, English Jersey and Russian Tagil. Among the Iberian breeds, a noticeable trace of zebu is observed in the Spanish Betizu, Rubia Gallega, Retinta and the Portuguese Brava de Lide. However, already at K = 6, the zebu trail becomes barely discernible in the Tagil, Brava de Lide and Retinta breeds. Analysing the structure of the *Bos indicus* breeds, we noted that the greatest influence of taurine cattle at K = 4 is expressed in the Sindi and Brahman breeds. In the work of Koufariotis et al. [59] devoted to the study of the genome of Brahman cattle, 892 genes were identified in regions with significant introgression of *Bos taurus*.

#### 2.3.6. African Breed Group (African Humpless *Bos taurus*, Humped *Bos indicus*, A.h. *Bos taurus* × Humped *Bos indicus*)

The influence of the African component on other breed groups becomes clear at values K = 10. In alignment with previous studies [14,34,40], Iberian cattle has a significant genetic component dating back to African taurines. The greatest contribution of the African component is demonstrated by the Vaca Canaria and Vaca Palmera breeds from the Canary Islands and Mallorquina from the Balearic Islands. The smallest contribution of this component is determined in *Bos indicus* and *Bos grunniens*. Among other breeds of the European cluster, a possible trace of African breeds is also demonstrated by breeds from Russia, including Kholmogory and Kostroma. In the Asian breed group, the greatest contribution of the African component is observed in two breeds from Russia (Kalmyk and Buryat), Gobi from Mongolia and Alatau from Kyrgyzstan. The presence of an African component in the genomes of the Russian [14] and Turano-Mongolian breeds has been considered in previous studies. Most of the African breeds show a high percentage of indicine ancestry, while the genetic structure of Gabu is almost completely devoid of this component. The Kuri breed belongs to the humpless cattle of West Africa [46]. However STRUCTURE analysis demonstrates that studied genomes contain an admixture of zebu. M’bororo belongs to the Zebu cattle of West Africa, while at K = 4 the contribution from *Bos taurus* is noticeable.

## 3. Discussion

As the methods of genotyping became more and more accessible, the number of studies devoted to the assessment of genetic diversity and the establishment of phylogenetic relationships of indigenous cattle breeds grew [3,4,5,6]. However, the questions of the origin of some local breeds remain unclear. Another important object of study is the domestic yak (*Bos grunniens*). Domestic yaks provide nomadic herders with valuable resources necessary for life. A large habitat of domestic yaks is in Russia. At the same time, to the best of our knowledge, large-scale studies involving large samples of yaks from Russia in the context of a wide list of *Bos taurus* breeds from different regions of the world and *Bos indicus* breeds have not yet been conducted. In order to determine the population genetic characteristics and to clarify the phylogenetic relationships of modern representatives of 155 cattle populations, a large body of data (10,250 individuals) was collected and analysed, including unique native cattle, 12 yak populations from Russia, Mongolia and Kyrgyzstan, as well as zebu breeds. Based on the results obtained, we provide a number of important clarifications regarding the genetic structure of the populations of native cattle and domestic yak.

### 3.1. Domestic Yak (Bos grunniens)

The populations of domestic yak from Mongolia have a component of Turano-Mongolian cattle breeds while components of European cattle breeds found in the Aikol yak from Kyrgyzstan (Figure 4). By using diagnostic markers, Qi et al. revealed introgression of the cattle genome in 22 of 29 yak populations, with an average frequency of 11.8%. mtDNA sequences and/or allelic variants of microsatellite markers specific to cattle were detected in 127 individuals [27]. Furthermore, the literature has examples of introgression of allelic variants of genes under domestication selection (for example, *MITF*) from cattle to yaks [60]. *MITF* is a growth factor, which participates in the regulation of melanocytes by controlling the synthesis of pigment [61]. As is known, during the domestication of cattle, the genes associated with coat colouration were under the strongest selection pressure [62]. Medugorac et al. found that 1.3% of the genes of the Mongolian yaks have been inherited from bovine ancestors [56]. The genes identified are involved in the development and functioning of the nervous system. While studying the origin of the Chinese domestic yak, Lai et al. recorded cases of gene introgression from Chinese cattle [63]. At K = 4, the genomic component of yaks is also present in some cattle breeds from different breed groups. Interestingly, two breeds from Africa, Gabú and Bafatá, demonstrate the largest contribution of that component. They, as well as the Muturu breed, which also contains a yak component, are grouped on a phylogenetic tree (Figure 1) next to the yak cluster rather than an African breed group. These breeds are less influenced by *Bos indicus*. Among the animals of the Asian breed group, a trace of possible introgression from the yak was also revealed, in particular in the Buryat and Aulie-Ata breeds. Wu et al. reported about the yak haplotypes introgression in genes involved in the response-to-hypoxia pathway (*EGLN1*, *EGLN2* and *HIF3a*) from yak to Tibetan cattle, which contributed to the adaptation of the latter to high altitude [60]. Cases of transfer of crucial haplotypes from related species that are already adapted to the local habitat are known in humans and animal species. For example, the crucial *EPAS1* haplotypes were transferred from Denisovans to Tibetans [64] and from Tibetan grey wolf to Tibetan Mastiff [65]. In studying the origin and adaptation of East Asian cattle, Chen et al. reported the introgression of 1.22% of the yak genome into the cattle breeds studied [57]. In addition, secondary introgression of yak mtDNA was determined in Diqing cattle [66]. It is worth noting that signs of selection for the *AQP5* gene in response to adaptation to high altitudes characterized by low temperatures have been observed in both yaks and cold-resistant Kholmogory cattle [10]. This may imply that selection takes place according to a similar scenario. Cases of mutual introgression of yak and cattle genes may probably be due to the history of their directed hybridization. The first hybrids of cattle and yak are thought to have been developed in China about 3000 years ago [67]. Yak hybrids with *Bos taurus* and *Bos indicus* cattle have larger body sizes, increased milk yield and are better adapted to a warm climate than yaks. At the same time, hybrid males (F1) are sterile, while females retain fertility [68]. Males restore their fertility only after four generations of backcrossing hybrid cows to parental bulls [69].

### 3.2. European Breed Group (Bos taurus)

Based on archaeological data, the authors of a number of studies suggest two main ways of agricultural cattle breeding in Europe: (1) the Danube way, along which Neolithic farmers moved north through the Central European plains, and (2) the Mediterranean way running along the Mediterranean coast [42,70]. Cattle accompanying people during migration interbred with local cattle and with local aurochs, which contributed to genetic diversity [71]. It is noted that cattle of the Middle East and Africa could leave their mark in the genomes of Mediterranean breeds [42]. Indirect confirmation of this hypothesis can be found in the results of structural analysis. At K = 10, the influence of the African group is observed in all Mediterranean breeds studied, and at K = 20, it remains clearly distinguishable in Italian Romagnola, Modicana and Cinisara. Mastrangelo et al. [14] found Mediterranean breeds, including Italian ones, being grouped closer to the African than to the Northern European breeds. In studying the mtDNA of breeds from central and southern Italy, Di Lorenzo et al. [72] found an increased frequency of occurrence of haplogroup T1, characteristic of breeds of Africa and the Middle East [73]. The results of Bayesian cluster analysis also indicate the presence of an Asian component in the genomes of some Italian breeds. In particular, at K = 12, the largest contribution of Asian ancestry among the Italian breeds was demonstrated by Podolian Podolica, Sicilian Modicana and Sicilian Cinisara. We have previously noted that, on the phylogenetic tree, the Kalmyk breed from the Asian breed group forms a cluster with the Italian ones. Additionally, although this clustering does not have sufficient bootstrap support, the results of structural analysis and Fst analysis provide some evidence in favor of them having a common ancestor. In studying different classifications of cattle breeds, we found that steppe breeds of Asia and Russia, including Kalmyk cattle, are often combined together with Italian breeds into a common breed group called “Steppe Cattle” [74]. This union can be observed in the classifications by Keller (1905) [75], Werner (1912) [76], Dechambre (1913) [77] and Bougler (1998) [78]. According to Keller [75], steppe cattle spread southeast from the Alps through Hungary, Romania, Turkey, Greece and southern Russia. In the east, this type has spread to Australia and may be the kin of South Siberian cattle. In the west, its area is considered to be Italy, where it was brought in the 6th century [75]. The results of molecular genetic studies also confirm the presence of kinship ties of the Kalmyk breed with both European and Asian breeds. The genetic proximity of Kalmyk cattle to the Grey Ukrainian and Mongolian breeds [13] has been described. Ukrainian Grey cattle, like some breeds from Italy, belong to the Podolian group [79]. Additionally, an opinion has previously been expressed about the possible European origin of Kalmyk cattle [30]. It is also interesting to note that in our work the Kalmyk cattle did not demonstrate any proximity to the Yakut cattle. At the same time, we do not exclude the presence of common ancestors in these breeds. According to the analysis of the Y-chromosome, one of the progenitors of Yakut cattle could be ancient steppe cattle [15]. At the same time, we note the possible presence of common ancestors in Yakut cattle with Turano-Mongolian breeds and breeds of the Podolian group, in particular Podolica. The joint clustering of Turano-Mongolian group breeds, including Kalmyk, with Podolian breeds has already been described [13]. Also note that at K = 12 among Italian breeds, Podolica demonstrated the greatest contribution of the Asian ancestry. At K = 20, the putative Asian component in Podolica showed commonality with the ancestor Buryat (Russia) and Khogorogo (Mongolia).

Among the Italian breeds considered, the Sicilian root (Cinisara and Modicana breeds) and Podolian (Romagnola, Marchigiana, Chianina and Podolica) can be distinguished [72]. Cinisara was long associated with the Modicana breed and only after 1995 was recognized as a separate breed [80]. Podolian breeds form a large group of grey cattle, usually with long horns, resulting from directed breeding [80]. Traditionally, it is believed that the ancestors of the Podolian cattle originate from Podolia (a region of modern Ukraine), from where they spread south to Anatolia and west to the Balkans and Italy (about 3–5 centuries AD) [72,81]. As an alternative hypothesis, Mediterranean migration from the Middle East to Central Italy [82] and possible hybridization with local wild aurochs [83] are considered. Chianina and Podolica are the oldest breeds of Italy. Marchigiana was bred by crossing native Podolian cattle with Chianina and Romagnola breeds in the late 19th–early 20th century in the Marche region. The history of origin is confirmed by the phylogenetic relationships observed on the dendrogram (Figure 1). The close genetic relationship of the three Podolian breeds Chianina, Marchigiana and Romagnola is also confirmed by the use of the same scheme for assessing the breeding value and similar breeding indices [81]. The Istrian breed, which came to the Istrian peninsula 2500 years ago, entered the group of Italian cattle. The peninsula is located in the northern part of Croatia, and a small part of it is shared by Slovenia and Italy. Territorial proximity may explain the joint clustering of this breed with the local breeds of Italy. The genetic proximity of the Istrian breed to Italian breeds was also demonstrated by Maretto et al. [81]. In addition, there are mentions of the introduction of Romagnola and Marchigiana bulls in the cattle population on the Istrian peninsula at the end of the 18th century [81].

The Podolian breed Hungarian Grey [84] appeared in a cluster represented mainly by Asian breeds and breeds from Russia. Senczuk et al. [85] mention the division of Grey cattle into two groups: (1) Asiatic grey Steppe cattle, which includes Hungarian Grey and (2) European breeds of the Podolian group, including Podolica, Chianina, Marchigiana and Romagnola. On the PCA plot, Hungarian Grey cattle occupied a position between the cluster of Asian breeds and the cluster with mostly Italian ones. There are several hypotheses about the origin of Hungarian Grey cattle. According to some sources [86] Hungarian Grey cattle arrived together with the Hungarian conquerors from the Podolic area in the 9th century. According to other sources [87], Hungarian Grey cattle descended from *Bos primigenius* and were domesticated on the territory of the Pannonian Basin during the reign of the Arpad dynasty. One of the first mentions of the long-horned Hungarian cattle can be found in a document of the 16th century [88]. It has been reported that the cattle trade was one of the main incomes of the country. Thus, Hungarian cattle have spread far beyond the borders of the state [88]. However, after the Second World War, a sharp decline in Hungarian Grey cattle population followed and the breed was on the verge of extinction. At the end of the 1950s, animals of the Kostroma dairy breed were involved in the breeding of Hungarian Grey [89]. By 1962, the Hungarian Grey cattle had a total of 200 purebred cows and six bulls [90]. Measures were taken to restore the population, but the reduction in livestock numbers could probably affect the intrabreed genetic diversity, and, as a result, the position of the breed on the phylogenetic tree relative to the other breeds. Some authors studying the genetic structure of cattle breeds noted that individuals of Hungarian Grey cattle form a separate group [72,86].

The beginning and spreading of pastoralism in Russia dates back to the 6th century AD. It was associated with the migrations of ancient Slavic tribes [91]. Over a long period of breed formation, local cattle developed a number of important adaptations to fare well in diverse natural and climatic conditions [92]. The genetic individuality of some cattle breeds in Russia has been demonstrated in a number of works [7,8,9]. In particular, rare allelic variants of genes responsible for adaptation, disease resistance and productivity were found in the Yakut, Kholmogory and Yaroslavl breeds [10,11,12]. As is known from archival documents, from the first quarter of the 18th century to the beginning of the 20th century, a variety of cattle breeds were imported into Russia, mainly from Western Europe [93]. However, opinions differ on the contribution of these breeds to the development of the Russian livestock population [94,95]. The origin of Russian cattle breeds has already been discussed in some major studies [3,13,14,15,16], however, consensus on some breeds has not been reached. On the phylogenetic tree and on the PCA plot, the Kholmogory breed was united together with the Dutch cattle. Their close genetic relationship is confirmed by the history of the development of the Kholmogory breed [33,92]. According to Bazhanov’s book dated 1867, this breed originates from Dutch cattle brought in by order of Peter the Great [94]. However, there is an opinion that Kholmogory cattle originated from crossing the local northern cattle with the Dutch ones [96,97]. Some authors consider these cattle as ancestors of an aboriginal breed that originates from a population of forest cattle [98]. The Red Gorbatov and Tagil breeds fell into a cluster of predominantly Asian breeds within the European cluster. Although in another study [13] the Red Gorbatov breed formed a branch within the British–Northern European cluster, we treat our result with caution and explain it by the absence of their direct ancestor breeds in our sample. At the origins of the formation of this breed was the local Prioksky Great Russian cattle, which was crossed with Tyrolean (Tux-Zillertal cattle) [99]. In addition, this clustering does not have sufficient bootstrap support. The same applies to the Tagil breed, which was bred by crossing the Ural native cattle with the Dutch and Kholmogory breeds, as well as partly with the Yaroslavl, Tyrolean and Swiss breeds [32,100]. Based on this, we expected to see Tagil cattle be closer to the Dutch and Kholmogory breeds. At the same time, we assume that over the long history of their development, the animals of the Red Gorbatov and Tagil breeds have evolved a number of adaptations that could substantially affect their genetic structure and distance them from the original breeds. In particular, the authenticity of the Red Gorbatov breed [3] was shown. In close proximity to the Kostroma breed were the Brown Swiss, Kyrgyz native and Alatau breeds. According to the literature, the Kostroma and Alatau breeds have extensive shared haplotypes with the Brown Swiss breed [3]. In the same work, Kostroma and Alatau were in the same cluster with breeds from southeastern France, Italy and Switzerland. The same cluster with the Kostroma breed, in addition to those listed, includes French Limousine, German Gelbvieh, Swiss Simmental and Spanish Pirenaica, which coincides with the clustering according to SNP analysis [13]. During the development of the Kostroma breed, bulls of the Algauz and Schwyz breeds were used, as well as animals of the Yaroslavl, Kholmogory, Simmental and Ayrshire breeds, as well as local cattle [101,102,103].

### 3.3. Asian Breed Group (Bos taurus)

Cattle domesticated on the Indian subcontinent or imported from the Fertile Crescent and Europe are considered by Decker et al. to be the ancestors of Asian cattle breeds [34]. These authors provide evidence of cattle exports from the Indian subcontinent to China and Southeast Asia [34]. Regarding the origin of East Asian cattle populations, Chen et al. [57] report the existence of three distinct ancestors: an early East Asian ancestor (*Bos taurus*), a later Eurasian ancestor (*Bos taurus*), and a new Chinese ancestor (*Bos indicus*) diverging from the Indian *Bos indicus*. Turano-Mongolian cattle belong to *Bos taurus*, but have morphological and genetic differences from European taurine cattle [57,104]. They are common in Northeast Asia and according to Xia et al. [105] include cattle from Russia (Buryatia, Yakutia), Kazakhstan, Mongolia, Korea, Japan and China (its northern and central part and Tibet). The greatest contribution of the Turano-Mongolian component can be traced in the Yakut breed, followed by Khogorogo from Mongolia and Buryat from Russia (Figure 4). The smallest contribution of this component among the Asian breed group was noted in Kyrgyz beef-type and Alatau from Kyrgyzstan. After the division of the Turano-Mongolian component into two parts (K = 20), the first part included the Buryat and Khogorogo breeds, and the second part - the Yakut breed. The genetic proximity of the Buryat breed from Russia and Khogorogo from Mongolia is due to the history of the Buryat people, who are closely related to Mongolians. Analysis of the Y-chromosome haplogroups showed a close genetic relationship of the Buryats with the Mongols [106]. As is known, the territory of ethnic Buryatia largely coincides with the territory of the country of Bargudzhin-Tokum [107], belonging to the Mongolian world. According to archaeological evidence, back in the Middle Ages, the people living in that territory were nomadic and seminomadic cattle breeders [108]. A book dated 1896 [109] describing the life of the rural population of the Irkutsk province presents cattle breeds bred, among others, by the Buryats. In total, nine groups were characterized, including local Buryat, Steppe Buryat, Mongolian and their hybrids. It is worth noting that, in Russia, until recently, the Buryat breed was considered extinct. However, about 200 Buryat cattle were recently found in remote areas of Mongolia and Inner Mongolia. Some of these animals were brought to Buryatia for further breeding [3].

The presence of the Asian component in the genomes of some breeds at low K values may indicate ancient hybridization events between the Asian breed group and other breeds due to human migration. One of these routes could run through the Mediterranean and connect Asia with Central Italy [82]. A number of studies provide evidence in favor of the hypothesis of a possible Asian origin of some Italian breeds, in particular the Podolian group. By analysing the mtDNA of some breeds of the Podolian group, Pellecchia et al. [82] found a genetic relationship of the Podolian group with breeds from the Balkans, Anatolia and the Middle East. The authors concluded that, genetically, Tuscan bovines are closer to the Middle Eastern than European gene pools, which is consistent with data on modern human populations from Tuscany, showing kinship with Anatolian and Middle Eastern human populations. The results obtained confirm the hypothesis of people and cattle migrating by sea from the Eastern Mediterranean region to the region corresponding to ancient Etruria (Tuscany, Central Italy). Senczuk et al. [71] and Xia et al. [105] provide data on the presence of mtDNA haplogroups characteristic of Turano-Mongolian populations in the genomes of some Italian breeds. Biometric research data also confirm that bulls belonging to *Bos taurus asiaticus* can be considered some of the progenitors of Podolian breeds [110].

The results of our structural analysis indicate the presence of the Asian component in the genomes of some Italian breeds. In particular, at K = 12 among Italian breeds, the greatest contribution of Asian ancestry was demonstrated by Podolian Podolica, Sicilian Modicana and Sicilian Cinisara. As noted above, at K = 20, the assumed Asian component in the Podolica breed reveals affinity with the ancestor Buryat (Russia) and Khogorogo (Mongolia). At the same time, on the PCA plot, Podolica formed a cluster with other Italian breeds, away from the Asian breed group. The exchange of cattle between Asia and Europe can also be traced in the genomes of Asian breeds. At K = 20, structural analysis of the Asian breed group shows that the genetic component of European breeds is clearly visible in most populations. The presence of the genetic component mentioned may be due to the export of European cattle to Asia, followed by hybridization of imported and local cattle. The results of cluster analysis at K = 15 and K = 20 by Decker et al. indicate the presence of an admixture of European taurine in the Mongolian breed and the Wagyu breed [34].

### 3.4. Iberian and Creole Breed Group (Bos taurus)

Domesticated cattle were brought by Christopher Columbus to the Caribbean island of Hispaniola from the Canary Islands during his second voyage across the Atlantic in 1493 [39]. Until 1512, Spanish colonists continued to import livestock, after which the animals were brought to America [40]. Cattle from the Canary Islands were descendants of Iberian cattle and could carry the genes of North African and Indic breeds [41,42]. Subsequently, cattle imported to America spread across the continent adapting to harsh climatic conditions and lack of food. After about 300 years, several other European breeds were introduced, as well as Indian Zebu breeds [43]. Later on, Creole cattle were displaced to more demanding areas [111]. Thus, the position of the Creole breed group on the phylogenetic tree is explained by the history of Creole cattle. Furthermore, the relationship of the Creole and Iberian groups was previously described in detail by Ginja et al. in their study of American Creole cattle [51]. The Creole cluster is followed by a group of African cattle and Zebu cattle. In another work, Ginja et al. looking into the origin and genetic diversity of Creole cattle described the influence of West African bulls on the genome of the Creole Caracu based on the analysis of mitochondrial DNA and Y-chromosome [44]. The influence of Zebu cattle on another Creole breed, Suriname, was noted in a project studying the dairy productivity of this breed [112].

### 3.5. Zebu Breed Group (Bos indicus)

The influence of *Bos indicus* is more widespread on the African and Creole breed group. In their work, MacHugh et al. noted that African cattle are characterized by a geographical gradient of indicine ancestry [113]. It was found that taurine cattle in West Africa have an average of 3.3% of indicine origin (from 0% to 19.9%). Moving from west to east and from south to central Africa, the proportion of introgression of *Bos indicus* genes increases on average to 56.9% (from 22.7% to 74.1%) [34]. Speaking about the Creole breed group, we note that according to published data, the introgression of the genetic component of zebu into the Creole cattle genome occurred in America [34]. At least two zebu introduction events have been described in the literature. Sevane et al. [111], referring to a work by Santiago, write about the hybridization of the Creole cattle with Indian cattle after 300 years of Creole populations spreading across the continent. Ajmone-Marsan et al. [114], in a study on the origin of cattle, consider the import of *Bos indicus* (mainly bulls) to Brazil in the 19th and 20th centuries [114]. The results of STR analysis [115], as well as data on mtDNA polymorphism [116,117] and Y-chromosome [118] of Creole cattle populations, confirm the events described. At K = 4 (Figure 4), all representatives of the Asian breed group demonstrate in their structure a trace of *Bos indicus*, best discernible in the Khogorogo breed from Mongolia. In a work by Yurchenko et al., the results of structural analysis (K = 3) obtained from genotyping data using SNP markers also demonstrate the contribution of indicine ancestors to the genome of Turano-Mongolian breeds [3]. The mtDNA haplotypes specific to zebu, have been found in cows of the Alatau breed [15], and in populations of Mongolian cattle [118]. According to some studies, the introgression of *Bos indicus* in the Mongolian and Kazakh breeds presumably occurred in the 2nd–7th centuries AD during the Silk Road period [118,119]. In studying the polymorphism of the Y-chromosome of cattle in Mongolia, Mannen et al. concluded that the introgression of zebu was secondary, since all the haplotypes discovered belonged to *Bos taurus* [118]. There is an opinion that during the same period, the hybridization of the local Asian cattle *Bos taurus* with African zebus may have occurred [120]. According to another version, Mediterranean cattle carrying alleles of African taurine and indicine were brought along the Silk Road, and subsequently were crossed with Far Eastern Asian cattle [34].

At K = 4 (Figure 4) impact of the *Bos indicus* genome to the European breed group can be noted, among others, in the Podolian cattle (Chianina, Romagnola, Podolica and Croatian Istrian). The introgression of *Bos indicus* genes into the Podolian cattle genome has been repeatedly noted in previous studies [71,121,122]. The genetic influence of zebu on the Podolian breed group may be mediated by ancient steppe cattle migrating from the southern steppe regions of Russia to southern, southeastern and central Europe more than a thousand years ago [123]. The trace of zebu in the genomes of the Sicilian breeds Cinisara and Modicana was also previously noted by Mastrangelo et al. [14]. The Jersey breed has also demonstrated influence from zebu. Based on Y-chromosomal microsatellite data, Kantanen et al. [15] reported a genetic relationship between the Jersey and Serbian Podolian cattle. These authors postulate that Jersey cattle may originate from ancient South Russian steppe cattle. In studying the molecular basis of coat colouration in steppe and Mediterranean grey cattle, Senczuk et al. [85] suggested that the phenotype of grey coat colouration and associated allelic variants of genes could have been inherited from zebu. In another work, Barbato et al. [122] identified the genomic regions that had been introduced from zebu to white cattle from Central Italy. These genomic regions contained genes responsible for body size and feed efficiency [122]. The ability to consume low-quality feed more efficiently could accelerate the adaptation of Italian breeds to limited food access. The results obtained are consistent with the previously described scenarios of cattle migration to Europe: the first way was from the Middle East with the capture of taurine cattle, which had already had an indicine introgression; the second way from West Africa to Spain included taurine cattle with no introgression of indicine [34]. Analysis of the mitochondrial DNA of the Spanish breed Lidia showed the existence of two ancestral lines of European and African [124].

### 3.6. African Breed Group (African Humpless Bos taurus, Humped Bos indicus, A.h. Bos taurus × Humped Bos indicus)

When studying the origin of African breeds, the genetic diversity of populations from pure *Bos taurus* to almost pure *Bos indicus* [47] was revealed. It was noted that indigenous African cattle were formed under the influence of taurine cattle adapted to local conditions and South Asian zebu cattle [125,126]. A significant role could also be played by mass replacements of cattle at the end of the 19th century after the panzootic plague [127]. According to the literature, all African cattle carry the mitochondrial DNA of taurine, which means they are not pure *Bos indicus* [47,73]. This fact may indicate that African zebu cattle and taurine-zebu hybrids originated from crossing African taurine females with zebu males from South Asia [114]. At the same time, the analysis of Y-chromosome haplotypes indicates the participation of both *Bos taurus* males and *Bos indicus* males [128,129]. According to the authors of the thematic works, the migration of zebu to Africa occurred from the Indian subcontinent through the Horn of Africa and was accompanied by two or more separate introductions [34,130]. The uniqueness of African breeds is also associated with possible hybridization events of domesticated taurine ancestors with the wild African aurochs [34]. The presence of African admixture in the genomes of breeds from southern Europe, including Iberian ones, may be a consequence of the migration of cattle through the Strait of Gibraltar during the Moorish conquests and occupation of the Iberian Peninsula (8th–13th centuries AD) [40,131]. Based on the results of mtDNA analysis of Bronze Age cattle from northern Spain, Anderung et al. [132] suggested an earlier contact between African and Iberian cattle. The greatest contribution of the African component was determined in Vaca Canaria and Vaca Palmera from the Canary Islands and Mallorquina from the Balearic Islands. As we could see previously, these breeds stood away from the general Iberian cluster on the phylogenetic tree, but were next to the African breeds Gabú, Bafatá and Muturu. The split-up of these breeds into separate branches may indicate the uniqueness of their gene pool. The presence of African ancestry in the Creole cattle genome is due to Iberian ancestors rather than the immediate involvement of African breeds [34,40]. There is also an alternative hypothesis that Creole cattle are directly influenced by African cattle imported to America [133] In support of this hypothesis, Ginja et al. [51] cite the results of mtDNA analysis, according to which the T1c haplogroup found in American Creoles is very rare in Iberia and can have been obtained directly from African cattle. The authors observed T1c lines in cattle from Guinea-Bissau and Angola and assumed that cows from these two countries could become a direct source of the T1c haplogroup.

To sum up, we note that most of the results obtained are in line with the history of breeds and similar studies, including those performed using SNP markers. The low bootstrap support observed for large breed-clusters on the phylogenetic tree could be expected. This pattern has already been noted earlier in the analysis of many populations [134], especially those represented by closely related breeds [35,51]. However, the results of the phylogenetic analysis (Figure 1) are consistent with those of other methods, including principal component analysis (Figure 2) and Bayesian cluster analysis (Figure 4).

At the same time, we consider it necessary to describe the possible limitations of the approaches used. These include a different number of samples in breeds, a small number of individuals of some breeds, and a reduction in the number of STR markers used. In addition, samples of the genetic material of some breeds involved in the analysis were obtained far from the places of the historical origin of the breed. And, depending on the location, they could undergo mating with other breeds in order to improve certain economically useful or adaptive qualities. Yurchenko et al. [3] showed that the differentiation of the Black Pied and Holstein breeds (Fst = 0.020) was lower than that between the samples of Herefords from Russia and Wales (Fst = 0.029). In addition, there are small-numbered local breeds in the sample, and the probability that they were crossed with other breeds to stabilize the number is high. This fact could also affect the joint clustering of some breeds. As is known from the history of the Hungarian Grey breed, in the middle of the 20th century, 1800 out of 2000–3000 cows were crossed with bull sires of the Kostroma breed [135]. It is also worth noting that when recreating the history of the origin of certain breeds, it is necessary to take into account the possible change in allele frequencies between modern populations and their ancestors. In comparing the samples of the genetic material of Kalmyk breed individuals obtained from modern animals and museum exhibits, Abdelmanova et al. [136] concluded that 83.33% of alleles in the museum samples are present in the current population. Verdugo et al. [137], studying the remains of ancient Middle Eastern cattle *Bos taurus* showed that the genomic signature of early populations may be hidden in modern individuals by a later admixture.

## 4. Materials and Methods

### 4.1. Sample Information and Microsatellite Data

The object of our study is the statistical analysis of an STR dataset of 155 native and transboundary cattle populations, including domestic yak and zebu populations. Genotyping was carried out using 12 highly stable and polymorphic microsatellites (*BM1824, BM2113, CSRM60, CSSM66, ETH3, ETH10, ETH225, ILSTS006, SPS115, TGLA53, TGLA122 and TGLA227*), which are included in the panel recommended by ISAG–FAO for studying the genetic diversity of cattle [31]. The description of the listed markers and their localization on cattle chromosomes are given in Table A2. The merged dataset consists of data from the following sources:

1. Original STR analysis data for 15 cattle populations and 12 yak populations provided by our laboratory team in the course of previous studies [8,17]. The dataset was uploaded in the ‘Mendeley Data’ public repository (V1, doi: 10.17632/9s6npfc744.1).
2. Open-access STR data of other cattle populations from different regions of the world were presented in the papers: Van de Goor et al. [138], data available in the electronic supplementary material of the article; Gargani et al. [139], allelic profiles were deposited in the Dryad database (http://doi.org/10.5061/dryad.d4500); Ginja et al. [51] data available in the Dryad, Dataset, https://doi.org/10.5061/dryad.5dv41ns43.

A total of 10,250 animals from 155 populations were studied, including 730 animals from 10 populations of the Asian breed group, 4728 animals from 36 populations of the European breed group, 1907 animals from 39 populations of the Iberian breed group, 1385 animals from 39 populations of the Creole breed group, 490 animals from 14 populations of the African breed group, 177 animals from 5 populations of the Zebu breed group (*Bos indicus*) and 833 animals from 12 populations of the Domestic yak breed group (*Bos grunniens*) (Table A3). Breed names in the paper are used according to original data.

### 4.2. Data Merging and Filtering

**Figure 5:**
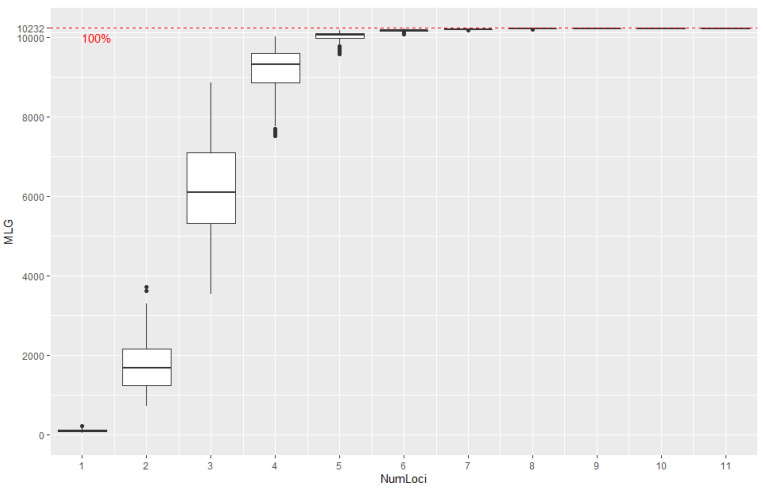
Genotype accumulation curve for all animals genotyped on 12 loci. The horizontal axis represents the number of loci randomly sampled without replacement up to (n−1) loci; the vertical axis shows the number of multilocus genotypes observed, the number of unique multilocus genotypes in the dataset. The red dashed line represents 100% of the total observed multilocus genotypes.

Before combining the data into one dataset, a data generalization procedure was performed. This procedure determines a nucleotide bias using the heat maps of the PopGenReport R package [140] and corrects the data. A total of 278 animals with a high percentage of missing data was removed. The minimum number of loci required for animal differentiation was determined using the poppr::as.genclone() function of the poppr R [141] package. Clone animals were removed using the popprclonecorrect() function of the same software package. The results of filtering the array from animal clones (10,250 instead 10,896) and establishing the optimal number of loci for research (11 loci) are shown in the graph (Figure 5). For each population, average values of standardized indices of association between all loci were calculated by the poppr::pair.ia() function. The results are in Table S3; the distribution diagram of average values of standardized indices of association among populations is shown in Figure S3.

### 4.3. Genetic Diversity Estimation

The calculations were performed in the R environment version 4.1.2. Mean number of alleles (Na) and effective number of alleles (Ne) were calculated using the Genalex package [142]. Allelic frequencies (AF) were calculated using the adegenet R package [143]. Allelic diversity parameters, observed (Ho) and expected (He) heterozygosity, inbreeding coefficient (Fis) and Hardy–Weinberg equilibrium (HWE) were calculated using the diveRsity R package [144]. PIC values were estimated for each locus and sample based on the number and frequency of alleles at the locus using the PopGenUtils package. Private alleles were calculated with the PopGenReport package. Nei’s pairwise Fst and their *p*-value were calculated by the pairwise.fst() function from the hierfstat R package [145] and visualized by the corrplot R package [146].

### 4.4. Phylogenetic Analysis

The genetic Da distance matrix [147] was calculated with the Hierfstat [145] package of the R environment. To reconstruct the phylogenetic tree, the neighbor-joining method was implemented using the Trex-online [148] program. The visualization of the tree in the form of a circular dendrogram with colour identification of clusters was performed using the tools of the iTOL application [149].

### 4.5. Principal Component Analysis

PCA was performed to investigate genetic relationships among studied breeds. The scripts used the ade4 and adegenet R-libraries. Calculations were carried out with the dudi.pca() function, input data were scaled out using the scaleGen() function with the “mean” method. Additional visualizations were performed on the adegraphics platform in R.

### 4.6. Bayesian Cluster Analysis

The population structure of 155 breed populations (10,250 animals) was studied using the STRUCTURE 2.3.4 software [150]. We tested K from 2 to 20 and K = 30, and each test was performed in 15 replicates using GNU Parallel [151]. We set the burnin length to 100,000, numreps to 300,000 and the admixture model and the INFERALPHA parameter to TRUE. Results for individuals were visualized using the PONG 1.5 software. We used the Evanno method [152] implemented in the Structure Harvester Software [153] to estimate the ΔK distribution. To handle the results from replicate analyses, we used Clumpp 1.1.2 (the LargeKGreedy method with 1000 repeats) [154]. For clarity, some breeds were merged in a cluster on the visualization step. Population results were visualized in the R environment.

## Figures and Tables

**Table 1:** Private breed-specific alleles by locus and sample.

Locus	Population	Allele	AF
Eth3	Siboney	101	0.040
Cssm66	Kyrgyz native	207	0.010
Cssm66	Curraleiro	209	0.031
ilsts006	Yak-Rus	293	0.003
ilsts006	Angola	301	0.069
ilsts006	Podolica	308	0.010
Tgla227	Yak-Rus	67	0.003
Tgla227	Gyr	121	0.017
Tgla227	Gyr	123	0.172
Tgla227	Gyr	125	0.034
Tgla122	Tagil	185	0.041
Sps115	Menoufis	232	0.023
Sps115	Khovd-Mongol	236	0.031
Eth225	Alistana	132	0.010
Tgla53	Siboney	194	0.020
Tgla53	Siboney	200	0.020
Csrm60	Kalmyk	86	0.010
Bm2113	Red Bororo	119	0.036
Bm2113	Yak-Rus	146	0.005
Bm1824	Sanga Tonga	172	0.060
Bm1824	Pantaneiro	196	0.042
Eth10	Aikol-Kyrgyz	203	0.010
Eth10	Sanmartinero	205	0.025

## Supplementary Materials

The following supporting information can be downloaded at: https://www.mdpi.com/article/10.3390/ijms24055061/s1.

## Appendix A

**Table A1:** Population parameters for the cattle samples studied.

Bread Name	N	Na	Ne	A	%	Ar	Ho	He	Fis	HWE
Holstein	2745	10.000	4.185	120	54.89	5.32	0.73	0.73	0.0032	0
Tagil	48.08	8.167	4.882	98	45.57	5.88	0.78	0.77	−0.09	5 × 10^−4^
Kholmogory	49.92	6.750	4.028	81	37.7	4.99	0.72	0.73	0.0031	0
Red Gorbatov	50	8.000	4.301	96	45.45	5.38	0.78	0.74	−0.0563	0.5788
Brown Swiss	42.75	8.083	4.527	97	45.89	5.83	0.77	0.76	−0.0184	0.7017
Kostroma	20	6.333	3.699	76	35.46	4.86	0.75	0.7	−0.0783	0.9291
Altai-Rus	12	3.417	2.684	41	19.55	3.22	0.64	0.58	−0.0947	0.0093
Kalmyk	52	9.250	4.985	111	51.68	6.21	0.77	0.78	0.0081	1 × 10^−4^
Aulie-Ata	40.58	9.500	4.850	114	53.68	6.18	0.78	0.77	−0.0055	0.1758
Alatau	49	9.583	5.014	115	53.8	6.4	0.77	0.77	−2 × 10^−4^	0.9043
Kyrgyz Beef-type	47	8.333	4.351	100	47.08	5.8	0.75	0.74	−0.0143	0.6891
Kyrgyz native	48.92	9.417	5.035	113	52.29	6.24	0.79	0.78	−0.015	0.9992
Yakut	98.83	5.667	2.872	68	31.9	4.15	0.63	0.61	−0.0228	0
Khogorogo	50	7.083	3.869	85	39.66	5.09	0.7	0.7	−0.0062	0.0042
Gobi	49	7.333	4.495	88	40.9	5.56	0.75	0.75	0.006	0.0071
Buryat	278.58	11.333	5.163	136	63.09	6.17	0.78	0.78	0.0026	0
Blonde D’Aquitaine	150	7.667	4.629	92	42.47	5.49	0.79	0.76	−0.0357	0
Belgian Blue	45	7.083	4.030	85	40.38	5.18	0.73	0.71	−0.0232	0.0167
Brandrood Cattle	38	5.917	3.302	71	33.48	4.47	0.65	0.64	−0.0054	0.3932
Charolais	77.92	7.750	4.260	93	43.13	5.34	0.7	0.71	0.0114	7 × 10^−4^
Dexter	291	7.667	3.693	92	42.61	4.84	0.68	0.71	0.0414	0
Dutch Friesian	40	7.250	4.344	87	40.53	5.32	0.69	0.73	0.051	0
Groningen Whiteheaded	20	5.083	2.781	61	29.83	4.16	0.62	0.6	−0.029	0.3151
Galloway	59	7.000	3.268	84	39.22	4.75	0.58	0.64	0.0998	0
Heck Cattle	39	4.500	2.941	54	25.47	3.79	0.62	0.61	−0.016	0
Hereford	141.42	6.917	3.834	83	39.37	4.9	0.7	0.72	0.0306	0
Limousin	157.5	8.167	4.398	98	45.65	5.49	0.75	0.75	0.0085	0
Dutch Belted	22	5.083	3.377	61	28.75	4.27	0.65	0.64	−0.0156	0.0849
Marchigiana	14	5.000	2.932	60	28.36	4.13	0.58	0.62	0.059	0.0641
Maas Rijn IJssel	37	6.167	4.035	74	34.67	4.93	0.77	0.73	−0.0617	0.0452
Scottisch Highlander	85	4.333	2.756	52	24.89	3.73	0.59	0.6	0.0139	0
Verbeterd Roodbont	38	7.167	4.372	86	39.22	5.4	0.72	0.73	0.0172	0.0781
Wagyu	15	4.667	2.780	56	27.11	4.03	0.65	0.62	−0.0534	0.4475
Waldviertler Blondvieh	45	6.000	3.459	72	34.04	4.7	0.67	0.65	−0.0382	0.0013
Chianina	28.25	5.667	2.834	68	32.16	4.17	0.6	0.61	0.0169	0.0513
Romagnola	22.67	5.750	3.336	69	32.51	4.56	0.7	0.66	−0.0577	0.5968
Modicana	48.08	8.250	4.148	99	47.44	5.46	0.66	0.75	0.1151	0
Hungarian Grey	60	6.750	3.570	81	37.84	4.79	0.68	0.7	0.0216	0
Istrian	44.08	8.083	4.224	97	44.87	5.64	0.74	0.74	−0.0039	0.7742
Podolica	49.75	9.500	4.631	114	52.5	6.06	0.74	0.77	0.0388	0
Cinisara	25.33	8.250	4.511	99	46.12	5.97	0.73	0.77	0.0441	0.2271
Texas Longhorn	78.33	8.333	4.722	100	47.48	5.8	0.74	0.76	0.0328	0
Florida Cracker	49.83	7.583	4.838	91	43.6	5.79	0.71	0.78	0.0797	0
Pineywoods	49	7.667	4.544	92	43.92	5.57	0.63	0.76	0.1656	0
Criollo Lechero Tropical	34.08	7.500	4.364	90	42.43	5.29	0.69	0.74	0.0656	0.6564
Criollo Poblano	37.5	8.417	4.831	101	47.99	5.95	0.72	0.78	0.0771	0.0026
Criollo Baja California	13	7.000	4.508	84	40.4	5.49	0.81	0.76	−0.0719	0.9832
Criollo Chihuahua	11.17	6.667	4.533	80	38.94	5.44	0.8	0.77	−0.0433	0.9136
Criollo Nayarit	22.5	8.083	5.011	97	46.59	6.08	0.78	0.79	0.0065	0.8536
Criollo Chiapas	19.08	7.583	4.786	91	43.02	5.78	0.77	0.77	0.0043	0.8864
Guabalá	23.33	5.583	3.352	67	31.53	4.35	0.61	0.6	−0.0079	0.1659
Guaymí	35.5	8.000	4.377	96	46.15	5.83	0.75	0.75	0.0011	0.9492
Suriname	50	9.667	5.343	116	54.56	6.5	0.79	0.79	0.0053	0.0896
Blanco Orejinegro	24.67	5.917	3.332	71	34.29	4.57	0.74	0.68	−0.0911	0.0353
Caqueteño	22.5	7.500	4.891	90	43.06	5.75	0.8	0.78	−0.0266	0.3225
Sanmartinero	19	6.167	3.802	74	35.84	4.85	0.74	0.72	−0.0266	0.9099
Romosinuano	17.08	4.750	3.362	57	27.8	4.21	0.66	0.67	0.0155	5 × 10^−4^
Costeño con Cuernos	22.92	5.667	3.763	68	33.13	4.71	0.75	0.71	−0.0546	0.3235
Chino Santandereano	21.67	7.500	4.365	90	43.19	5.67	0.74	0.76	0.0286	0.4263
Velasquez	22.17	6.750	4.269	81	38.99	5.35	0.72	0.75	0.0283	0.5645
Lucerna	14.17	6.500	3.998	78	37.36	5.15	0.67	0.72	0.0761	0.585
Hartón del Valle	20.67	8.000	4.960	96	45.45	6.08	0.8	0.79	−0.0213	1
Criollo Limonero	42.83	7.000	4.007	84	40.61	5.23	0.74	0.71	−0.0532	0.0348
Criollo Ecuatoriano	40.92	8.667	4.973	104	49.62	6.02	0.74	0.78	0.0416	0.0784
Criollo Macabeo	24.58	8.167	4.785	98	45.91	5.97	0.8	0.76	−0.0422	0.0579
Caracú	74	8.750	4.329	105	49.27	5.68	0.7	0.74	0.0561	0
Crioulo Lageano	38.83	9.667	5.396	116	55.47	6.44	0.74	0.76	0.026	0.5209
Curraleiro	49.92	9.417	5.129	113	53.29	6.12	0.69	0.76	0.0956	0
Mocho Nacional	47.83	9.417	5.166	113	53.83	6.18	0.78	0.78	0.0064	0
Pantaneiro	47.83	9.250	5.146	111	52.88	6.34	0.77	0.78	0.0101	0.0028
Criollo Yacumeño	22.33	6.167	3.146	74	36.49	4.46	0.72	0.72	0.1622	0.0373
Criollo Uruguayo	41.92	5.833	3.307	70	33.56	4.47	0.67	0.67	−0.0011	0
Pampa Chaqueño	49.92	8.417	4.856	101	49.03	6.09	0.77	0.78	0.0159	0.8347
Criollo Pilcomayo	36	7.750	4.994	93	44.31	6	0.81	0.78	−0.0374	0.3587
Criollo Argentino	49.75	6.167	3.566	74	35.01	4.57	0.7	0.69	−0.0149	0
Criollo Patagónico	34.42	5.333	3.279	64	30.13	4.2	0.62	0.66	0.0637	0
Criollo Patagónico Chileno	33.92	7.250	4.661	87	41.23	5.52	0.73	0.77	0.0453	1 × 10^−4^
Senepol	19.58	5.583	3.435	67	32.14	4.51	0.74	0.69	−0.0734	0.3307
Criollo Cubano	44.33	7.417	4.659	89	42.78	5.55	0.78	0.76	−0.0222	0
Siboney	49.75	8.167	4.807	98	46.34	5.94	0.72	0.76	0.0484	0
Betizu	43.33	7.083	4.035	85	39.61	5.18	0.66	0.72	0.0814	1 × 10^−4^
Monchina	50	8.250	4.505	99	46.91	5.75	0.75	0.76	0.0117	0.1833
Lidia	44.83	6.417	3.670	77	36.28	4.66	0.62	0.68	0.0903	0
Alistana	50	7.417	3.825	89	42.41	5.09	0.68	0.71	0.0433	0
Sayaguesa	48	7.000	4.379	84	40.04	5.35	0.7	0.74	0.0557	0
Tudanca	50	7.500	4.327	90	42.52	5.48	0.7	0.72	0.0311	0
Asturiana de los Valles	50	9.083	4.991	109	51.59	6.19	0.74	0.77	0.0314	0.052
Asturiana de las Montañas	50	7.500	4.185	90	42.54	5.39	0.71	0.73	0.0348	0
Retinta	50	8.167	4.751	98	46.42	5.78	0.73	0.76	0.0371	0
Morucha	50	7.833	4.564	94	44.86	5.69	0.72	0.76	0.0418	2 × 10^−4^
Avileña	49.92	8.167	4.834	98	46.08	5.8	0.72	0.76	0.0484	0
Pirenaica	50	7.583	4.196	91	42.61	5.43	0.72	0.74	0.0253	0
Rubia Gallega	50	7.583	4.266	91	43.18	5.42	0.7	0.72	0.0306	0
Serrana de Teruel	50	8.000	4.895	96	44.84	5.87	0.76	0.78	0.0259	0
Parda de Montaña	50	7.833	3.907	94	44.51	5.53	0.72	0.72	0.0012	0.0105
Bruna de los Pirineos	43.67	7.417	4.195	89	42.64	5.28	0.73	0.73	−0.0068	0.3086
Pasiega	49.92	7.917	4.488	95	45.02	5.71	0.73	0.74	0.0134	0
Berrenda en Colorado	39.83	8.000	5.143	96	44.94	6.04	0.77	0.79	0.0257	0.252
Berrenda en Negro	26.08	5.750	3.144	69	32.97	4.42	0.62	0.65	0.0511	6 × 10^−4^
Marismeña	49.25	5.667	2.786	68	32.5	4.19	0.59	0.61	0.0339	0
Pajuna	37.92	7.500	4.432	90	42.46	5.51	0.71	0.74	0.0337	0.002
Negra Andaluza	49.33	8.667	4.631	104	48.25	5.88	0.67	0.74	0.0903	0
Menorquina	41.92	5.917	3.212	71	33.4	4.31	0.67	0.65	−0.0185	0
Mallorquina	49.33	4.583	2.807	55	26.13	3.65	0.62	0.6	−0.0299	0
Vaca Canaria	47.25	8.000	4.648	96	44.52	5.65	0.73	0.76	0.0477	0.4643
Vaca Palmera	49.92	5.917	3.417	71	33.06	4.46	0.64	0.65	0.0014	0
Alentejana	38	5.917	3.400	71	33.8	4.59	0.63	0.68	0.0765	0
Arouquesa	69	8.167	4.693	98	45.13	5.7	0.73	0.76	0.0314	0
Barrosã	69	6.500	3.691	78	36.8	4.9	0.68	0.68	−0.0055	0
Brava de Lide	42.92	5.917	3.448	71	33.98	4.45	0.6	0.65	0.0802	0
Cachena	51	7.750	4.238	93	43.64	5.41	0.72	0.71	−0.007	3 × 10^−4^
Garvonesa	39	6.417	3.577	77	36.72	4.63	0.72	0.67	−0.0777	0
Marinhoa	46	6.417	3.732	77	35.86	4.86	0.73	0.71	−0.0304	0
Maronesa	46.83	7.000	3.661	84	39.6	5.05	0.7	0.7	−3 × 10^−4^	0
Mertolenga	63.5	8.167	4.325	98	46.22	5.47	0.67	0.75	0.1037	0
Minhota	49.92	7.917	4.324	95	44.41	5.66	0.8	0.74	−0.0861	0.7863
Mirandesa	53.92	5.833	2.987	70	33.67	4.12	0.65	0.65	−0.0099	0
Preta	59.92	8.000	4.056	96	44.91	5.37	0.68	0.7	0.0351	0.1376
Ramo Grande	43.83	8.167	4.198	98	45.7	5.53	0.71	0.74	0.0305	0.4001
Aberdeen Angus	61.75	6.500	3.819	78	36.38	4.79	0.68	0.72	0.0538	0
British White Cattle	29.83	6.000	3.595	72	34.31	4.62	0.71	0.69	−0.0214	0.0018
Jersey	19.92	5.250	3.121	63	30.82	4.31	0.67	0.65	−0.0275	0.0306
Shorthorn	27	6.083	3.044	73	35.1	4.56	0.64	0.62	−0.0365	0.3411
Simmental	19	6.917	3.453	83	39.31	5.09	0.64	0.69	0.0642	0.0327
Gelbvieh	26	6.500	3.762	78	36.93	5.11	0.71	0.7	−0.0102	0.7697
Baladi	97.25	11.333	5.362	136	63.76	6.57	0.74	0.77	0.0435	0
Menoufis	20.58	8.083	4.868	97	46.62	5.76	0.68	0.73	0.0688	0.9526
Landim	11.75	7.250	4.615	87	41.15	5.77	0.75	0.74	−0.0152	0.9905
Angola	28.75	6.500	3.504	78	37.48	4.85	0.71	0.69	−0.0196	0.0022
Bafatá	19.75	5.333	2.778	64	30.94	3.94	0.63	0.6	−0.0618	09882
Gabú	25	5.750	2.843	69	32.86	4.13	0.62	0.61	−0.0166	0.1537
Ankole-Watusi	45.75	5.833	3.051	70	32.38	4.3	0.6	0.62	0.0262	0
Sanga Tonga	24.08	6.833	3.768	82	39.25	4.94	0.62	0.71	0.1195	0
Pokot	94.83	10.917	5.076	131	62.05	6.52	0.74	0.78	0.051	0
Eastern Shorthorn Zebu	42.58	8.333	4.050	100	47.78	5.36	0.64	0.7	0.0897	0
Sokoto Gudali	17.75	7.417	3.865	89	43.02	5.4	0.66	0.72	0.0821	0.0021
Red Bororo	13.67	6.833	3.917	82	39.18	5.44	0.73	0.733	−0.0049	0.9882
Muturu	20.33	6.667	3.810	80	38.2	5.06	0.63	0.7	0.0911	0.8777
Kuri	12.33	5.917	3.867	71	34.34	4.83	0.7	0.7	2 × 10^−4^	0.8407
Gyr	26.83	6.333	3.523	76	35.53	4.55	0.67	0.68	0.0226	0.0584
Brahman	34.5	7.250	3.623	87	41.38	4.86	0.69	0.69	0.0088	0.0017
Sindi	9.67	5.583	3.482	67	31.44	4.69	0.74	0.66	−0.1322	0.9976
Guzerat	14.67	5.083	2.804	61	28.8	4.13	0.62	0.62	0.004	0.9818
Nelore	84.33	7.917	3.004	95	44.79	4.55	0.63	0.63	0.0014	0
Yak-Rus	323	6.750	3.086	81	38.81	3.92	0.6	0.65	0.0769	0
Okinsk-Rus	45	4.250	2.765	51	24.44	3.71	0.63	0.6	−0.0437	0
Yak-Mongol	31	6.000	3.344	72	34.42	4.65	0.64	0.67	0.0392	0
Bay-Beldyr-Rus	36	4.500	2.663	54	26.3	3.41	0.62	0.6	−0.0287	0
Khovd-Mongol	49	5.833	3.079	70	33.83	4.09	0.63	0.64	0.0067	0
Malchyn-Rus	59	4.083	2.883	49	23.66	3.48	0.58	0.63	0.0688	0
Agrosoyuz-Rus	58	4.417	2.959	53	25.27	3.53	0.67	0.64	−0.0392	0
Mogen-Buren-Rus	58	3.833	2.695	46	22.26	3.49	0.66	0.61	−0.0781	0
Adargan-Rus	33	4.000	2.725	48	23.2	3.42	0.63	0.61	−0.0345	0
Aryg-Khem-Rus	59.75	4.417	2.811	53	25.09	3.51	0.57	0.61	0.057	0
Aikol-Kyrgyz	49	4.750	2.918	57	28.02	3.94	0.64	0.63	−0.023	0

Notation: N: the average number of individuals genotyped by the locus; Na: mean number of alleles; Ne: effective number of alleles; A: the number of alleles per sample; %: the average percentage of the total number of alleles observed in the locus; Ar: the mean allelic richness across loci; Ho and He: the observed and expected heterozygosity, correspondently; HWE: the *p*-value for the chi-square testing the Hardy–Weinberg equilibrium; Fis: inbreeding coefficient.

**Table A2:** Description of microsatellite markers.

Locus and Source Reference	Position on Chromosome	Repeating Sequences	Sequences Forward (F) and Inverse (R) Primers	Length of Amplicons (bp)
BM1824 [155]	D1S34	(GT)n	F: GAGCAAGGTGTTTTTCCAATC R: CATTCTCCAACTGCTTCCTTG	176–188
BM2113 [156]	D2S26	(CA)n	F: GCTGCCTTCTACCAAATACCC R: CTTCCTGAGAGAAGCAACACC	124–146
CSRM60 [157]	D10S5	(AC)n	F: AAGATGTGATCCAAGAGAGAGGCA R: AGGACCAGATCGTGAAAGGCATAG	91–117
CSSM66 [155]	D14S31	(AC)n	F: AATTTAATGCACTGAGGAGCTTGG R: ACACAAATCCTTTCTGCCAGCTGA	177–203
ETH3 [158]	D19S2	(GT)nAC(GT)6	F: GAACCTGCCTCTCCTGCATTGG R: ACTCTGCCTGTGGCCAAGTAGG	100–128
ETH10 [158]	D5S3	(AC)n	F: GTTCAGGACTGGCCCTGCTAACA R: CCTCCAGCCCACTTTCTCTTCTC	206–222
ETH225 [159]	D9S2	(TG)4CG(TG)(CA)n	F: GATCACCTTGCCACTATTTCCT R: ACATGACAGCCAGCTGCTACT	139–157
ILSTS006 [160]	D7S8	(GT)n	F: TGTCTGTATTTCTGCTGTGG R: ACACGGAAGCGATCTAAACG	279–297
SPS115 [161]	D15	(CA)nTA(CA)6	F: AAAGTGACACAACAGCTTCACCAG R: AACCGAGTGTCCTAGTTTGGCTGTG	247–261
TGLA53 [162]	D16S3	(TG)6CG(TG)4(TA)n	F: GCTTTCAGAAATAGTTTGCATTCA R: ATCTTCACATGATATTACAGCAGA	151–187
TGLA122 [162]	D21S6	(AC)n(AT)n	F: AATCACATGGCAAATAAGTACATAC R: CCCTCCTCCAGGTAAATCAGC	136–182
TGLA227 [162]	D18S1	(TG)n	F: GGAATTCCAAATCTGTTAATTTGCT R: ACAGACAGAAACTCAATGAAAGCA	76–104

**Table A3:** Information on cattle samples.

Breed	n	n*	Location of Sample	Reference	Number for Structure
*Asian breed group*					
Aulie-Ata	42	41	Talas region, Talas District, (Kyrgyzstan)	*Svishcheva et al.* [8]	9
Alatau	49	49	Chui region, Zhayilsky district (Kyrgyzstan)	*Svishcheva et al.* [8]	10
Buryat	286	279	Khuvsgul aimag (Mongolia)	*Svishcheva et al.* [8]	16
			Inner Mongolia (China)	*Svishcheva et al.* [8]	16
			Buryatia Republic, Dzhidinsky District (Russia)	*Svishcheva et al.* [8]	16
Gobi	50	49	South Gobi aimag (Mongolia)	*Svishcheva et al.* [8]	15
Kalmyk	54	52	Kalmykia republic, Yustinsky district (Russia)	*Svishcheva et al.* [8]	8
Khogorogo	50	50	Khuvsgul aimag (Mongolia)	*Svishcheva et al.* [8]	14
Kyrgyz Beef-type	48	47	Chui region, Panfilovsky district (Kyrgyzstan)	*Svishcheva et al.* [8]	11
Kyrgyz native	49	49	Naryn region, At-Bashinsky District (Kyrgyzstan)	*Svishcheva et al.* [8]	12
Yakut	99	99	Yakutia republic (Russia)	*Svishcheva et al.* [8]	13
Wagyu	20	15	Japan	*Van de Goor et al.* [138]	33
*European breed group*					
Brown Swiss	129	44	Kostroma region, Kostroma district (Russia)	*Svishcheva et al.* [8]	5
			Berne (Germany)	*Gargani et al.* [139]	5
			Switzerland (sampled in Mexico)	*Martínez et al.* [133]	5
Holstein	3023	2746	Moscow region (Russia)	*Svishcheva et al.* [8]	1
			The Netherlands	*Van de Goor et al.* [138]	1
			The Netherlands (sampled in Portugal)	*Martínez et al.* [133]	1
Kostroma	20	20	Kostroma Region, Kostroma district, (Russia)	*Svishcheva et al.* [8]	6
Kholmogory	50	50	Komi republic, Inta (Russia)	*Svishcheva et al.* [8]	3
Red Gorbatov	50	50	Nizhny Novgorod region, Pavlovsky district (Russia)	*Svishcheva et al.* [8]	4
Tagil	49	49	Perm region, Oktyabrsky District (Russia)	*Svishcheva et al.* [8]	2
Istrian	45	45	*Institut national de la recherche agronomique, INRA* (France)/Giessen (Germany)	*Gargani et al.* [139]	39
Chianina	36	30	*Institut national de la recherche agronomique, INRA* (France)/Piacenza (Italy)	*Gargani et al.* [139]	35
Cinisara	30	26	Catania (Italy)/Van Hall Larenstein, University of Applied Sciences (Netherlands)	*Gargani et al.* [139]	41
Modicana	50	49	Catania (Italy)	*Gargani et al.* [139]	37
Podolica	50	50	Campobasso(Italy)	*Gargani et al.* [139]	40
Romagnola	32	24	*Institut national de la recherche agronomique, INRA*(France)/Piacenza(Italy)	*Gargani et al.* [139]	36
Blonde D’Aquitaine	165	150	France	*Van de Goor et al.* [138]	17
Belgian Blue	51	45	Belgium	*Van de Goor et al.* [138]	18
Brandrood Cattle	41	38	Netherlands	*Van de Goor et al.* [138]	19
Charolais	86	78	France	*Van de Goor et al.* [138]	20
			France (sampled in Portugal)	*Martínez et al.* [133]	20
Dexter	471	291	Republic of Ireland	*Van de Goor et al.* [138]	21
			UK (sampled in USA)	*Ginja et al.* [51]	21
Dutch Friesian	42	40	Netherlands	*Van de Goor et al.* [138]	22
Groningen Whiteheaded	24	20	Netherlands	*Van de Goor et al.* [138]	23
Galloway	88	59	Scotland	*Van de Goor et al.* [138]	24
Heck Cattle	39	39	Germany	*Van de Goor et al.* [138]	25
Hereford	150	142	The UK	*Van de Goor et al.* [138]	26
			UK (sampled in Argentina, Mexico, USA)	*Martínez et al.* [133]	26
Limousin	173	158	France	*Van de Goor et al.* [138]	27
			France (sampled in Portugal)	*Martínez et al.* [133]	27
Dutch Belted	24	22	Netherlands	*Van de Goor et al.* [138]	28
Marchigiana	17	14	Italy	*Van de Goor et al.* [138]	29
Maas Rijn IJssel	41	37	Netherlands	*Van de Goor et al.* [138]	30
Scottish Highlander	118	85	Scotland	*Van de Goor et al.* [138]	31
Verbeterd Roodbont	42	38	Netherlands	*Van de Goor et al.* [138]	32
Waldviertler Blondvieh	45	45	Austria	*Van de Goor et al.* [138]	34
Aberdeen Angus	62	62	UK (sampled in Argentina & USA)	*Martínez et al.* [133]	120
British White Cattle	30	30	UK (sampled in USA)	*Martínez et al.* [133]	121
Jersey	20	20	UK (sampled in USA)	*Martínez et al.* [133]	122
Shorthorn	28	27	UK (sampled in USA)	*Martínez et al.* [133]	123
Gelbvieh	26	26	Germany (sampled in USA)	* Ginja et al. * [51]	125
Simmental	19	19	Switzerland (sampled in USA)	* Ginja et al. * [51]	124
Hungarian Grey	60	60	Vienna	*Gargani et al.* [139]	38
*Iberian breed group*					
Alentejana	38	38	Portugal	*Martínez et al.* [133]	107
Arouquesa	70	69	Portugal	*Martínez et al.* [133]	108
Barrosã	69	69	Portugal	*Martínez et al.* [133]	109
Brava de Lide	43	43	Portugal	*Martínez et al.* [133]	110
Cachena	51	51	Portugal	*Martínez et al.* [133]	111
Garvonesa	39	39	Portugal	*Martínez et al.* [133]	112
Marinhoa	46	46	Portugal	*Martínez et al.* [133]	113
Maronesa	47	47	Portugal	*Martínez et al.* [133]	114
Mertolenga	64	64	Portugal	*Martínez et al.* [133]	115
Minhota	50	50	Portugal	*Martínez et al.* [133]	116
Mirandesa	54	54	Portugal	*Martínez et al.* [133]	117
Preta	60	60	Portugal	*Martínez et al.* [133]	118
Ramo Grande	44	44	Portugal (Azores Islands)	*Martínez et al.* [133]	119
Alistana	50	50	Spain	*Martínez et al.* [133]	84
Asturiana de las Montañas	50	50	Spain	*Martínez et al.* [133]	88
Asturiana de los Valles	50	50	Spain	*Martínez et al.* [133]	87
Avileña	50	50	Spain	*Martínez et al.* [133]	91
Berrenda en Colorado	40	40	Spain	*Martínez et al.* [133]	98
Berrenda en Negro	30	27	Spain	*Martínez et al.* [133]	99
Betizu	49	44	Spain	*Martínez et al.* [133]	81
Bruna de los Pirineos	50	46	Spain	*Martínez et al.* [133]	96
Marismeña	50	50	Spain	*Martínez et al.* [133]	100
Monchina	50	50	Spain	*Martínez et al.* [133]	82
Morucha	50	50	Spain	*Martínez et al.* [133]	90
Negra Andaluza	50	50	Spain	*Martínez et al.* [133]	102
Pajuna	38	38	Spain	*Martínez et al.* [133]	101
Parda de Montaña	50	50	Spain	*Martínez et al.* [133]	95
Pasiega	50	50	Spain	*Martínez et al.* [133]	97
Pirenaica	50	50	Spain	*Martínez et al.* [133]	92
Retinta	50	50	Spain	*Martínez et al.* [133]	89
Rubia Gallega	50	50	Spain	*Martínez et al.* [133]	93
Sayaguesa	48	48	Spain	*Martínez et al.* [133]	85
Serrana de Teruel	50	50	Spain	*Martínez et al.* [133]	94
Lidia	50	48	Spain	*Martínez et al.* [133]	83
Tudanca	50	50	Spain	*Martínez et al.* [133]	86
Mallorquina	50	50	Spain (Balearic Islands)	*Martínez et al.* [133]	104
Menorquina	50	44	Spain (Balearic Islands)	*Martínez et al.* [133]	103
Vaca Canaria	50	48	Spain (Canary Islands)	*Martínez et al.* [133]	105
Vaca Palmera	50	50	Spain (Canary Islands)	*Martínez et al.* [133]	106
*Creole breed group*					
Criollo Argentino	50	50	Argentina	*Martínez et al.* [133]	75
Criollo Patagónico	35	35	Argentina	*Martínez et al.* [133]	76
Criollo Yacumeño	30	25	Bolivia	* Ginja et al. * [51]	71
Caracú	74	74	Brazil	*Martínez et al.* [133]	66
Crioulo Lageano	39	39	Brazil	*Egito et al.* [115]	67
Curraleiro	50	50	Brazil	*Egito et al.* [115]	68
Mocho Nacional	50	49	Brazil	*Egito et al.* [115]	69
Pantaneiro	48	48	Brazil	*Egito et al.* [115]	70
Criollo Patagónico Chileno	38	35	Chile	* Ginja et al. * [51]	77
Blanco Orejinegro	25	25	Colombia	*Martínez et al.* [133]	54
Caqueteño	25	24	Colombia	*Martínez et al.* [133]	55
Chino Santandereano	25	22	Colombia	*Martínez et al.* [133]	59
Costeño con Cuernos	25	23	Colombia	*Martínez et al.* [133]	58
Hartón del Valle	22	21	Colombia	*Martínez et al.* [133]	62
Lucerna	23	15	Colombia	*Martínez et al.* [133]	61
Romosinuano	25	18	Colombia	*Martínez et al.* [133]	57
Sanmartinero	24	20	Colombia	*Martínez et al.* [133]	56
Velasquez	25	23	Colombia	*Martínez et al.* [133]	60
Criollo Cubano	50	46	Cuba	*Martínez et al.* [133]	79
Siboney	50	50	Cuba	*Martínez et al.* [133]	80
Criollo Ecuatoriano	46	42	Ecuador	*Martínez et al.* [133]	64
Criollo Macabeo	25	25	Ecuador	*Vargas et al. 2016* [163]	65
Criollo Baja California	20	14	Mexico	*Martínez et al.* [133]	47
Criollo Chiapas	30	20	Mexico	*Martínez et al.* [133]	50
Criollo Chihuahua	16	12	Mexico	*Martínez et al.* [133]	48
Criollo Lechero Tropical	46	37	Mexico	* Ginja et al. * [51]	45
Criollo Nayarit	24	24	Mexico	*Martínez et al.* [133]	49
Criollo Poblano	42	38	Mexico	*Martínez et al.* [133]	46
Guabalá	25	24	Panama	*Martínez et al.* [133]	51
Guaymí	36	36	Panama	*Martínez et al.* [133]	52
Criollo Pilcomayo	36	36	Paraguay	*Martínez et al.* [133]	74
Pampa Chaqueño	50	50	Paraguay	*Martínez et al.* [133]	73
Senepol	22	20	Saint Croix Island (Caribe)	* Ginja et al. * [51]	78
Suriname	50	50	Suriname	* Ginja et al. * [51]	53
Criollo Uruguayo	43	43	Uruguay	*Martínez et al.* [133]	72
Florida Cracker	50	50	USA	* Ginja et al. * [51]	43
Pineywoods	50	49	USA	* Ginja et al. * [51]	44
Texas Longhorn	80	80	USA	*Martínez et al.* [133]	42
Criollo Limonero	48	43	Venezuela	*Martínez et al.* [133]	63
*African breed group*					
Angola	29	29	Angola	* Ginja et al. * [51]	129
Baladi	101	100	Egypt	* Ginja et al. * [51]	126
Menoufis	27	22	Egypt	* Ginja et al. * [51]	127
Bafatá	20	20	Guinea	* Ginja et al. * [51]	130
Gabú	25	25	Guinea	* Ginja et al. * [51]	131
Eastern Shorthorn Zebu	47	45	Kenya	* Ginja et al. * [51]	135
Pokot	104	99	Kenya	* Ginja et al. * [51]	134
Ankole-Watusi	46	46	Lake Victoria (sampled in the USA)	* Ginja et al. * [51]	132
Landim	13	12	Mozambique	* Ginja et al. * [51]	128
Kuri	21	13	Nigeria	* Ginja et al. * [51]	139
Muturu	21	21	Nigeria	* Ginja et al. * [51]	138
Red Bororo	14	14	Nigeria	* Ginja et al. * [51]	137
Sokoto Gudali	22	19	Nigeria	* Ginja et al. * [51]	136
Sanga Tonga	36	25	Zambia	* Ginja et al. * [51]	133
*Zebu breed group (Bos indicus)*					
Guzerat	15	15	India (sampled in Brazil)	*Martínez et al.* [133]	143
Nelore	89	87	India (sampled in Brazil)	*Martínez et al.* [133]	144
Sindi	11	10	India (sampled in Brazil)	*Martínez et al.* [133]	142
Brahman	41	36	India (sampled in Mexico & USA)	*Martínez et al.* [133]	141
Gyr	36	29	India (sampled in Mexico)	*Martínez et al.* [133]	140
*Domestic yak breed group (Bos grunniens)*					
Adargan-Rus	60	33	Ovyursky District (Russia)	*Oyun et al.* [17,18]	153
Agrosoyuz-Rus	58	58	Kyzyl (Russia)	*Oyun et al.* [17,18]	151
Altai-Rus	15	12	Kosh-Agachsky District (Russia)	*Oyun et al.* [17,18]	7
Aikol-Kyrgyz	50	49	Jeti-Oguz District (Kyrgyz Republic)	*Oyun et al.* [17,18]	155
Aryg-Khem-Rus	60	60	Barun-Khemchiksky District (Russia)	*Oyun et al.* [17,18]	154
Bay-Beldyr-Rus	56	56	Mongun-Tayginsky District (Russia)	*Oyun et al.* [17,18]	148
Khovd-Mongol	49	49	Khovd (Mongolia)	*Oyun et al.* [17,18]	149
Malchyn-Rus	60	59	Mongun-Tayginsky District (Russia)	*Oyun et al.* [17,18]	150
Mogen-Buren-Rus	60	58	Mongun-Tayginsky District (Russia)	*Oyun et al.* [17,18]	152
Yak-Mongol	31	31	Mongolia	*Oyun et al.* [17,18]	147
Okinsk-Rus	46	45	Okinsky District (Russia)	*Oyun et al.* [17,18]	146
Yak-Rus	336	323	Russia	*Oyun et al.* [17,18]	145
**Total:**	**11,174**	**10,250**			

Notation: n*: the number of individuals after data generalization procedure.
