# Supplementary material for: Genetic Structure Analysis of 155 Transboundary and Local Populations of Cattle (Bos taurus, Bos indicus and Bos grunniens) Based on STR Markers
Source: Int J Mol Sci. 2023 Mar 6;24(5):5061. doi: 10.3390/ijms24055061 (PMC10003406; doi:10.3390/ijms24055061)
Supplement: Supplementary file 1 [file ijms-24-05061-s001.zip › Figure S1.pdf]

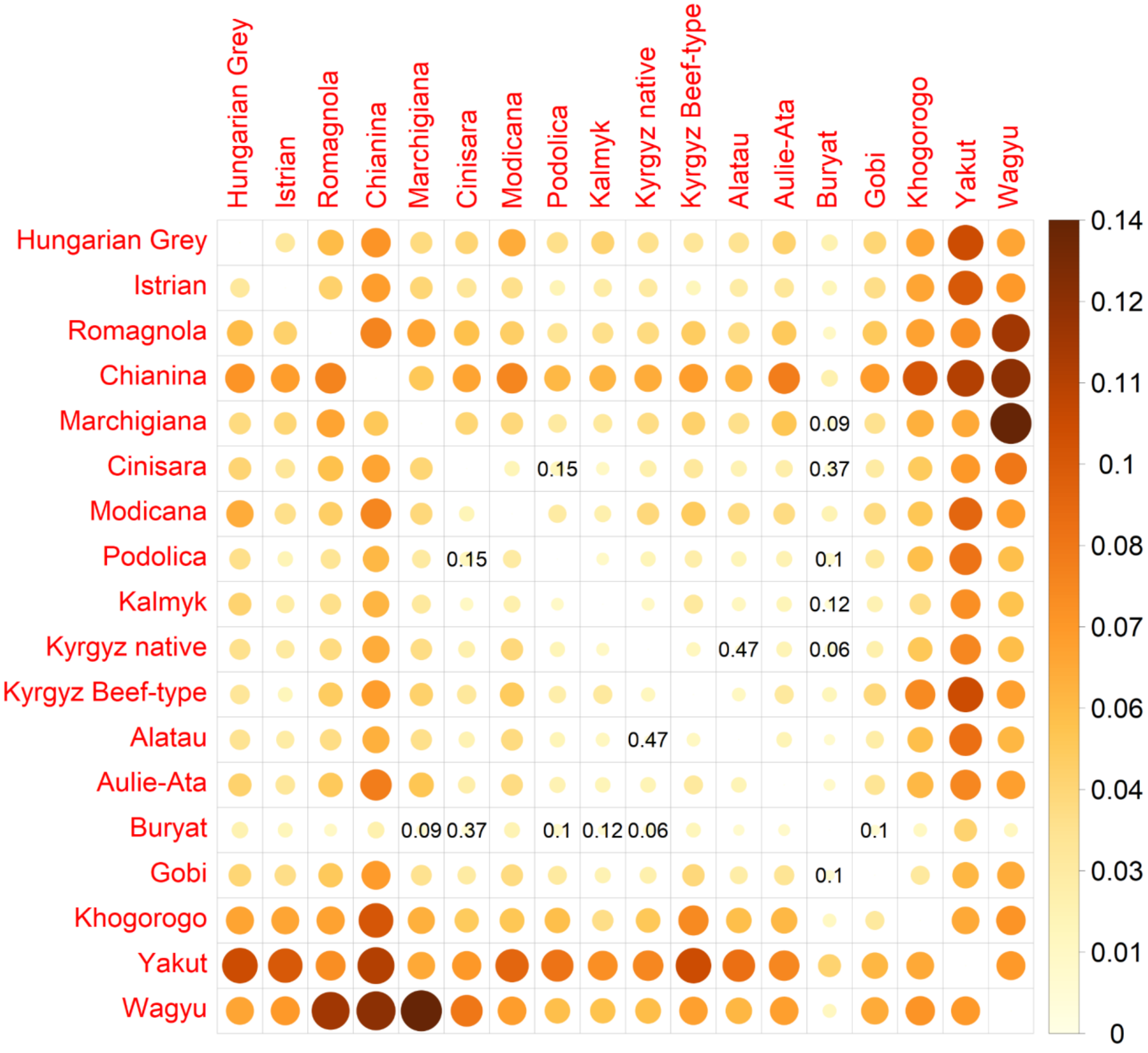

Figure S1: Correlation matrix for pairwise Fst values. Numbers added to the cells with p-value more than 0.05.
