# Supplementary material for: Genetic Structure Analysis of 155 Transboundary and Local Populations of Cattle (Bos taurus, Bos indicus and Bos grunniens) Based on STR Markers
Source: Int J Mol Sci. 2023 Mar 6;24(5):5061. doi: 10.3390/ijms24055061 (PMC10003406; doi:10.3390/ijms24055061)
Supplement: Supplementary file 1 [file ijms-24-05061-s001.zip › Figure S2. Cluster analysis of 155 cattle populations for K from 2 to 20 and K=30.pdf]

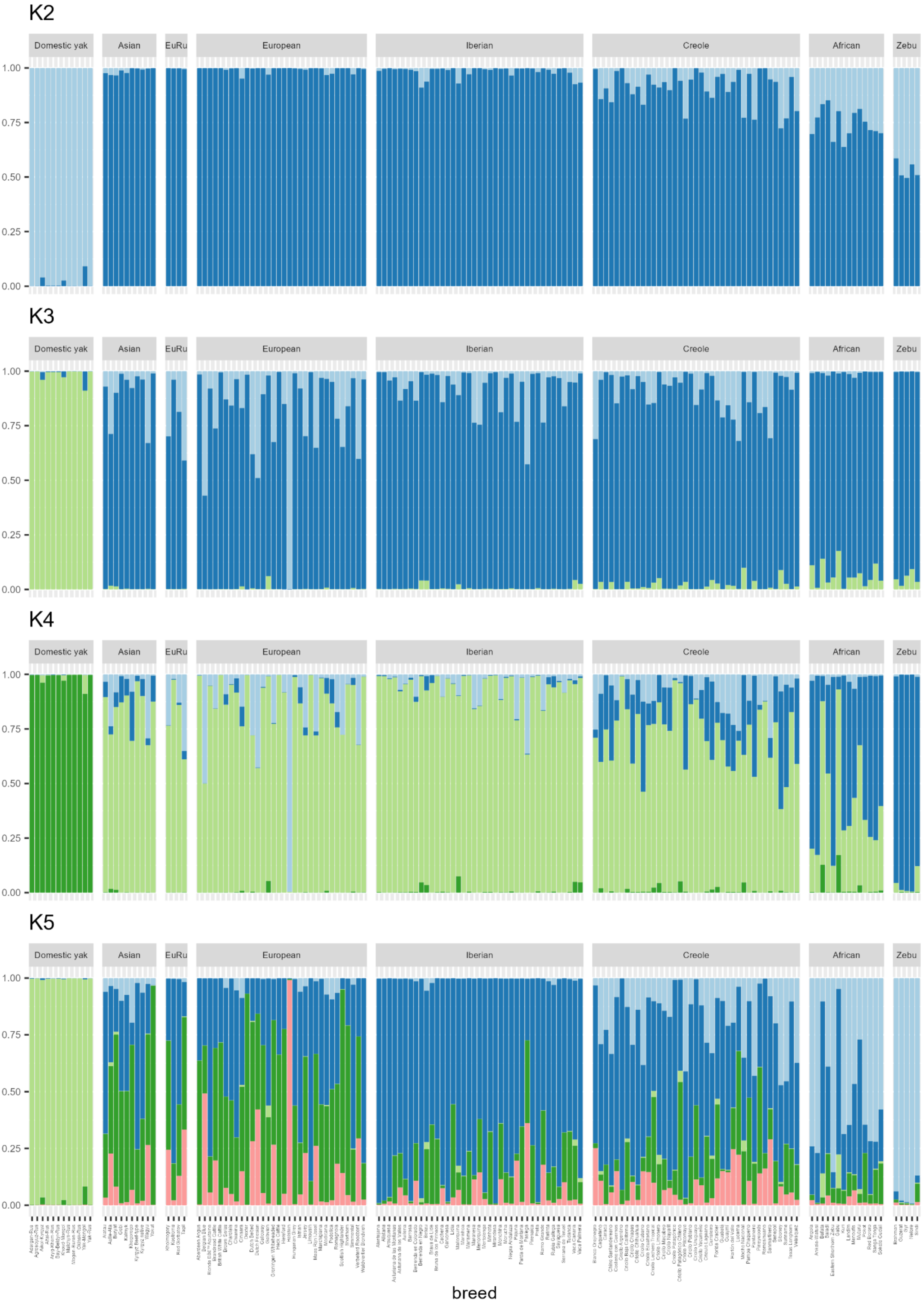
K2
K3
K4
K5
Domestic yak
Asian
EuRu
European
Iberian
Creole
African
Zebu
1.00
0.75
0.50
0.25
0.00
breed

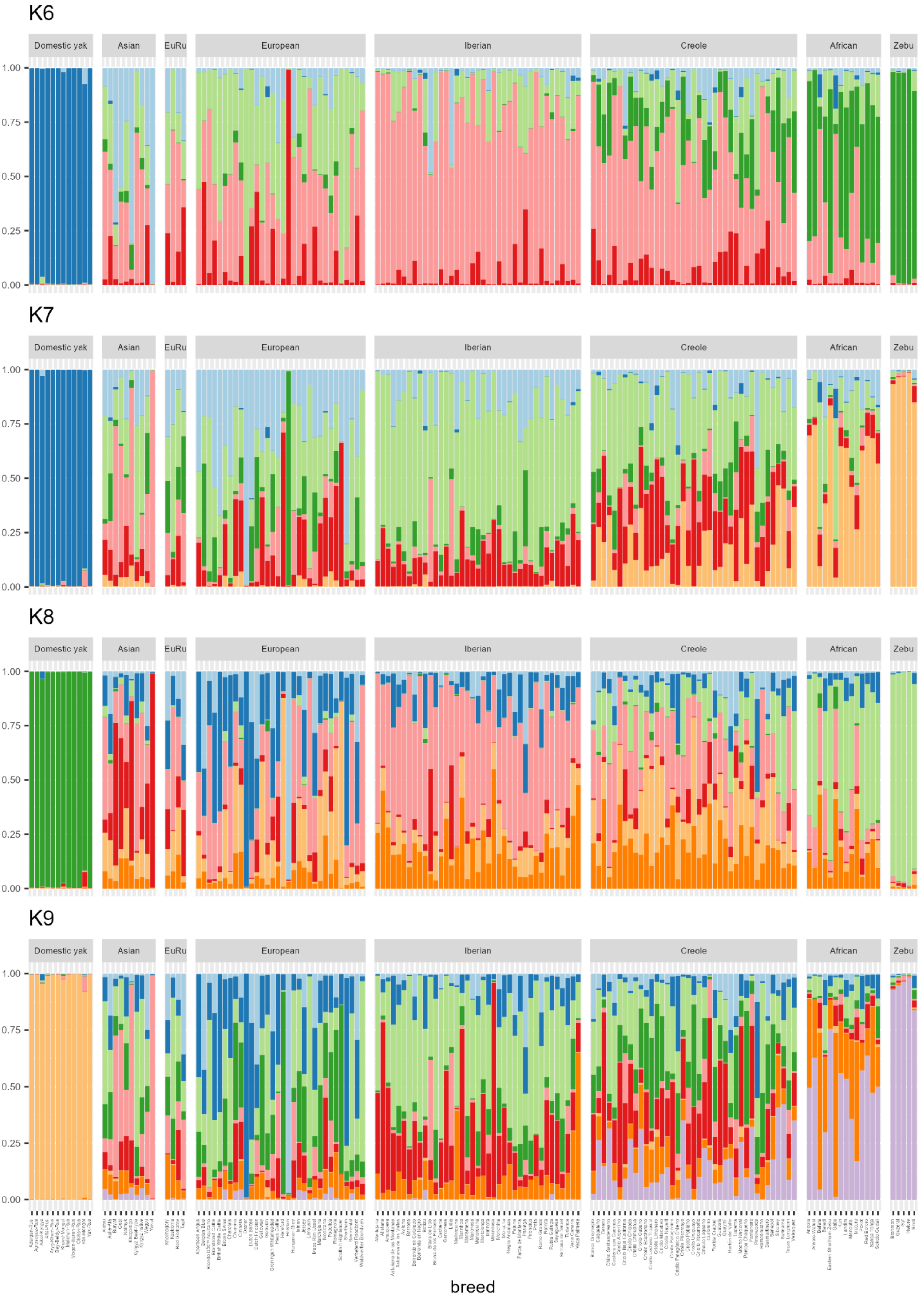
K6
K7
K8
K9
Domestic yak
Asian
EuRu
European
Iberian
Creole
African
Zebu
1.00
0.75
0.50
0.25
0.00
breed

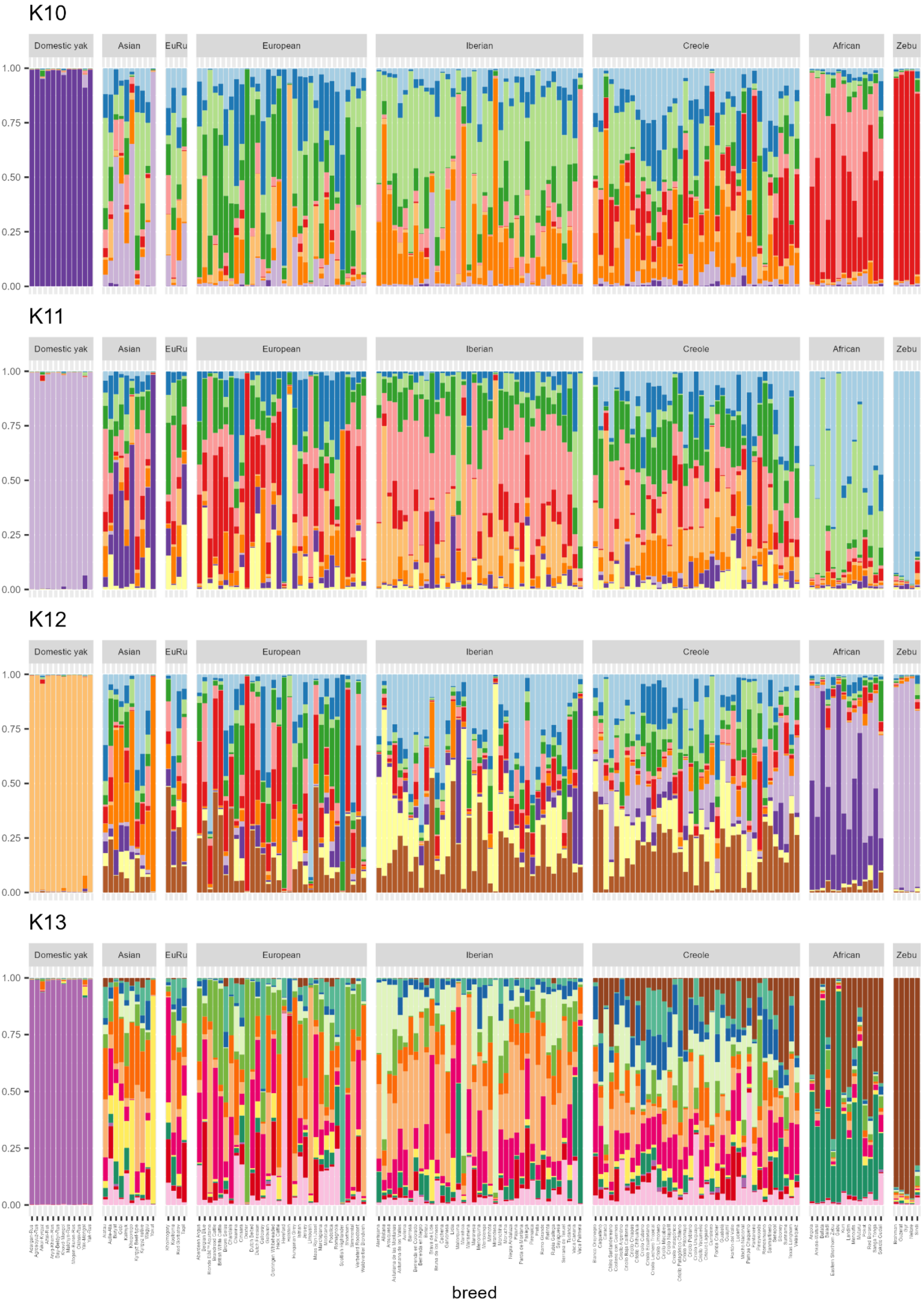
K10
K11
K12
K13
Domestic yak
Asian
EuRu
European
Iberian
Creole
African
Zebu
1.00
0.75
0.50
0.25
0.00
breed

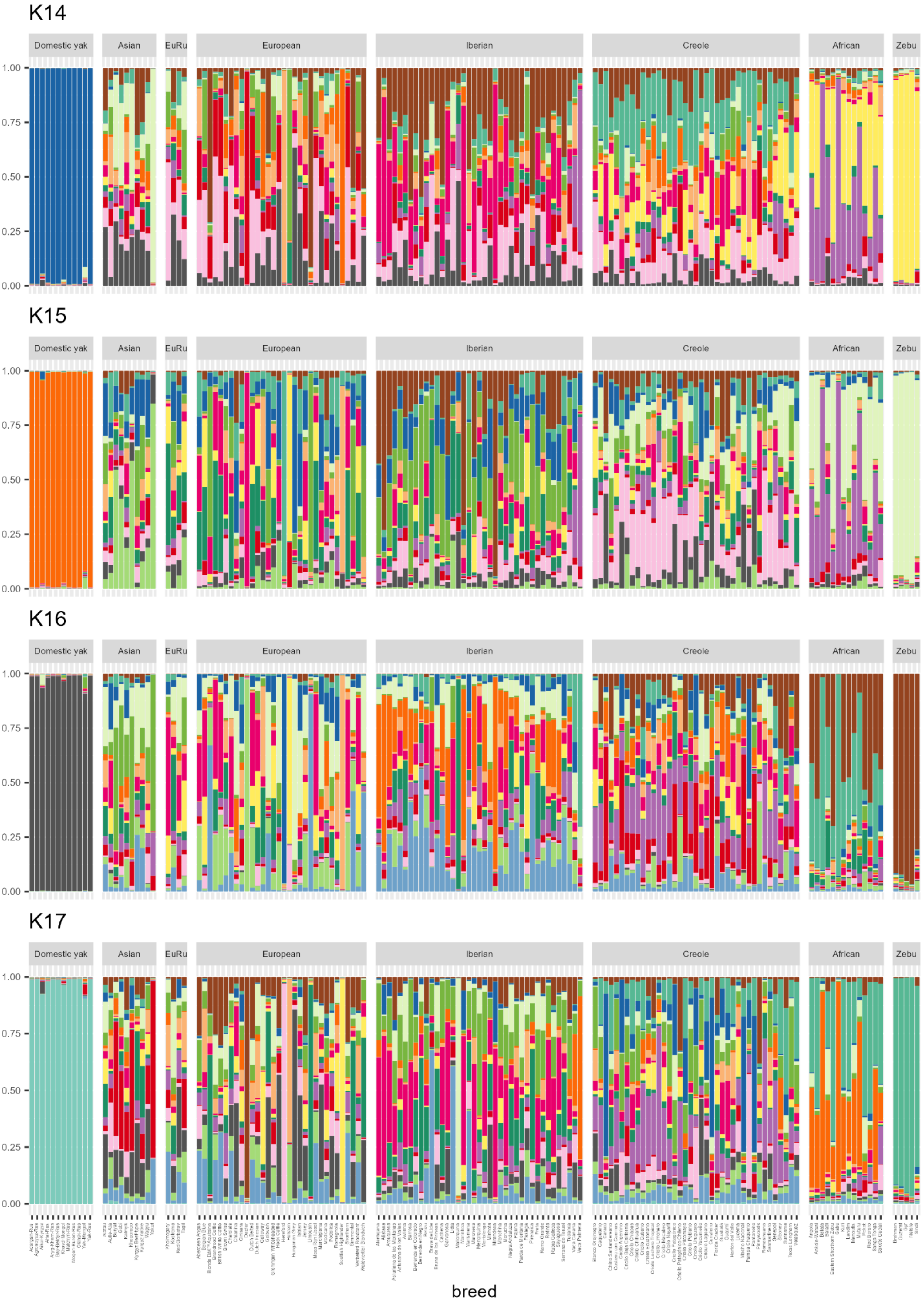
K14
K15
K16
K17
Domestic yak
Asian
EuRu
European
Iberian
Creole
African
Zebu
1.00
0.75
0.50
0.25
0.00
breed

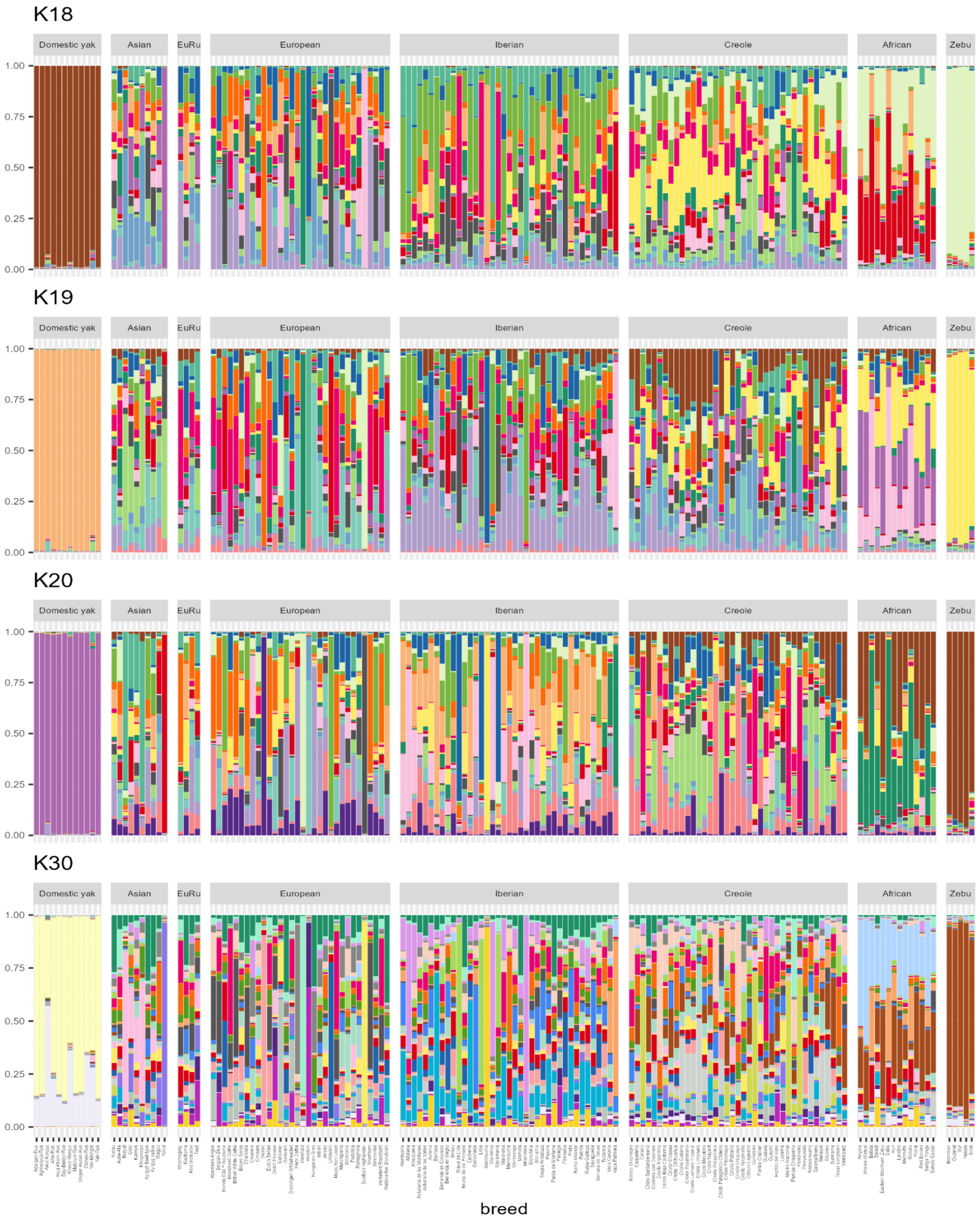

Figure S2: Cluster analysis of 155 cattle populations calculated from microsatellite data in STRUCTURE. Each individual breed is represented by a vertical bar divided into K colors according to the number of supposed ancestral populations. The size of the color segment is proportional to the contribution of a particular ancestral population to the genome of the breed in question. Results are presented for K from 2 to 20 and K=30.
