# Supplementary material for: Genetic Structure Analysis of 155 Transboundary and Local Populations of Cattle (Bos taurus, Bos indicus and Bos grunniens) Based on STR Markers
Source: Int J Mol Sci. 2023 Mar 6;24(5):5061. doi: 10.3390/ijms24055061 (PMC10003406; doi:10.3390/ijms24055061)
Supplement: Supplementary file 1 [file ijms-24-05061-s001.zip › Table S3 and Figure S4.pdf]

Table S3: Average values of standardized Indices of association between all loci for each population. Calculations were performed by the poppr::pair.ia () function.

Population name	LD, Standardized Index of Association between all loci
Limousin	0
Siboney	0.001
Criollo Limonero	0.001
Gabú	0.002
Landim	0.002
Yak-Rus	0.002
Verbeterd Roodbont	0.003
Hereford	0.003
Charolais	0.003
Asturiana de las Montañas	0.004
Belgian Blue	0.004
Holstein	0.004
Baladi	0.004
Cachena	0.004
Criollo Cubano	0.004
Blonde D'Aquitaine	0.004
Cinisara	0.005
Kalmyk	0.005
Chianina	0.005
Rubia Gallega	0.005
Pirenaica	0.005
Shorthorn	0.005
Barrosã	0.005
Arouquesa	0.005
Bruna de los Pirineos	0.005
Agrosoyuz-Rus	0.005
Aryg-Khem-Rus	0.006
Kyrgyz native	0.006
Hartón del Valle	0.006
Morucha	0.006
Minhota	0.006
Pampa Chaqueño	0.006
Criollo Yacumeño	0.006
Modicana	0.006
Asturiana de los Valles	0.007
Alentejana	0.007
Avileña	0.007
Vaca Canaria	0.008
Caracú	0.008

Alatau	0.008
Dexter	0.008
Maronesa	0.008
Hungarian Grey	0.008
Eastern Shorthorn Zebu	0.008
Caqueteño	0.009
Criollo Patagónico	0.009
Monchina	0.009
Marinhoa	0.01
Kholmogory	0.01
Tagil	0.01
Aberdeen Angus	0.01
Texas Longhorn	0.011
Nelore	0.011
Vaca Palmera	0.011
Criollo Pilcomayo	0.012
Adargan-Rus	0.012
Parda de Montaña	0.012
Podolica	0.012
Menoufis	0.012
Aikol-Kyrgyz	0.012
Pokot	0.013
Brava de Lide	0.013
Menorquina	0.014
Guaymí	0.014
Lidia	0.014
Senepol	0.015
Malchyn-Rus	0.015
Okinsk-Rus	0.015
Brown Swiss	0.015
Romagnola	0.015
Curraleiro	0.015
Pantaneiro	0.016
Yakut	0.017
Istrian	0.018
Retinta	0.018
Criollo Ecuatoriano	0.018
Guzerat	0.018
Red Gorbatov	0.018
Sayaguesa	0.019
Brahman	0.019
Suriname	0.02
Waldviertler Blondvieh	0.021
Gelbvieh	0.021
British White Cattle	0.021
Criollo Poblano	0.021

Mertolenga	0.021
Heck Cattle	0.021
Mocho Nacional	0.021
Maas Rijn IJssel	0.022
Preta	0.022
Wagyu	0.022
Tudanca	0.023
Alistana	0.024
Bay-Beldyr-Rus	0.024
Lucerna	0.024
Buryat	0.025
Berrenda en Colorado	0.025
Sindi	0.025
Simmental	0.025
Angola	0.025
Costeño con Cuernos	0.026
Pasiega	0.026
Criollo Uruguayo	0.027
Bafatá	0.029
Groningen Whiteheaded	0.03
Ankole-Watusi	0.031
Mogen-Buren-Rus	0.031
Kyrgyz Beef-type	0.031
Criollo Lechero Tropical	0.031
Guabalá	0.032
Criollo Nayarit	0.032
Chino Santandereano	0.033
Khovd-Mongol	0.034
Blanco Orejinegro	0.034
Brandrood Cattle	0.034
Velasquez	0.035
Romosinuano	0.035
Mirandesa	0.036
Criollo Argentino	0.036
Ramo Grande	0.038
Serrana de Teruel	0.038
Criollo Chiapas	0.038
Mallorquina	0.039
Gyr	0.039
Dutch Friesian	0.039
Gobi	0.04
Khogorogo	0.041
Dutch Belted	0.041
Red Bororo	0.041
Pajuna	0.041
Criollo Macabeo	0.042

Aulie-Ata	0.042
Betizu	0.044
Crioulo Lagueano	0.044
Jersey	0.045
Garvonesa	0.045
Scottisch Highlander	0.045
Marismeña	0.049
Florida Cracker	0.051
Kostroma	0.051
Criollo Chihuahua	0.051
Criollo Patagónico Chileno	0.052
Criollo Baja California	0.064
Marchigiana	0.067
Altai-Rus	0.074
Galloway	0.076
Sokoto Gudali	0.082
Pineywoods	0.086
Muturu	0.086
Sanmartinero	0.088
Sanga Tonga	0.088
Negra Andaluza	0.1
Yak-Mongol	0.103
Kuri	0.123
Berrenda en Negro	0.203

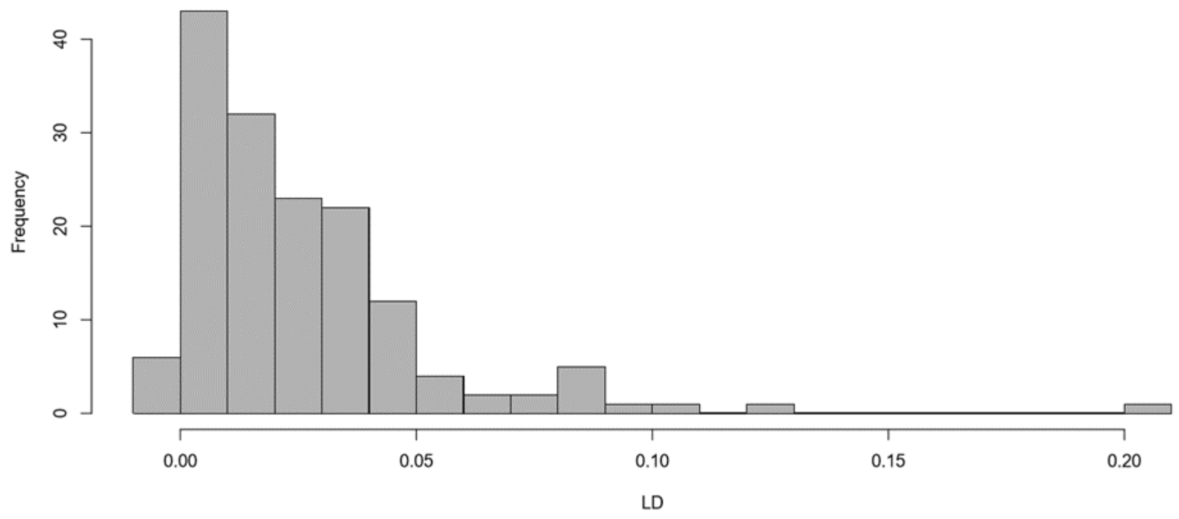

Figure S4. Distribution of Average values of standardized Indices of association among populations.
